# Engineering of Conserved Sequence Motif 1 Residues in Halohydrin Dehalogenase HheC Simultaneously Enhances Activity, Stability, and Enantioselectivity

**DOI:** 10.1021/acscatal.5c00819

**Published:** 2025-03-13

**Authors:** Sophie Staar, Miquel Estévez-Gay, Felix Kaspar, Sílvia Osuna, Anett Schallmey

**Affiliations:** † Institute for Biochemistry, Biotechnology and Bioinformatics, Technische Universität Braunschweig, Spielmannstr. 7, Braunschweig 38106, Germany; ‡ Institut de Química Computacional i Catàlisi (IQCC), Departament de Química, Universitat de Girona, c/Maria Aurèlia Capmany 69, Girona, Catalonia 17003, Spain; § Chair of Bioprocess Engineering, Institute of Biotechnology, Faculty III Process Sciences, Technische Universität Berlin, Ackerstraße 76, Berlin 13355, Germany; ∥ ICREA, Passeig Lluís Companys 23, Barcelona, Catalonia 08010, Spain; ⊥ Braunschweig Integrated Center of Systems Biology (BRICS), Technische Universität Braunschweig, Rebenring 56, Braunschweig 38106, Germany; # Center of Pharmaceutical Engineering (PVZ), Technische Universität Braunschweig, Franz-Liszt-Str. 35a, Braunschweig 38106, Germany

**Keywords:** halohydrin dehalogenase, protein engineering, epoxide ring opening, dehalogenation, spectrophotometric assay, quantum mechanics, molecular dynamics

## Abstract

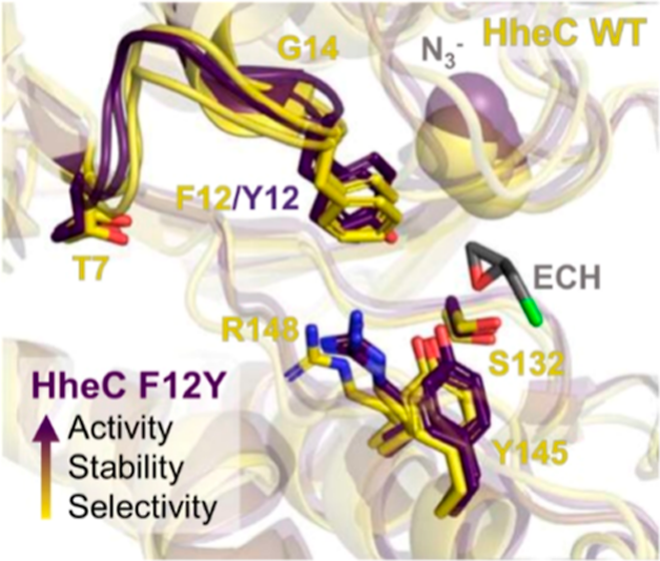

Halohydrin dehalogenases (HHDHs) are powerful enzymes for the asymmetric diversification of oxyfunctionalized synthons. They feature two characteristic sequence motifs that distinguish them from homologous short-chain dehydrogenases and reductases. Sequence motif 1, carrying a conserved threonine, glycine, and a central aromatic residue, lines the nucleophile binding pocket of HHDHs. It could therefore impact nucleophile binding and presumably also the activity of the enzymes. However, experimental evidence supporting this theory is largely missing. Herein, we systematically studied the mutability of the three conserved motif 1 residues as well as their resulting impact on enzyme activity, stability, and selectivity in two model HHDHs: HheC from *Agrobacterium radiobacter* AD1 and HheG from *Ilumatobacter coccineus*. In both HheC and HheG, the conserved threonine and glycine tolerated mutations to only structurally similar amino acids. In contrast, the central aromatic (i.e., phenylalanine or tyrosine) residue of motif 1 demonstrated much higher variability in HheC. Remarkably, some of these variants featured drastically altered activity, stability, and selectivity characteristics. For instance, variant HheC F12Y displayed up to 5-fold increased specific activity in various epoxide ring opening and dehalogenation reactions as well as enhanced enantioselectivity (e.g., an *E*-value of 74 in the azidolysis of epichlorohydrin compared to *E* = 22 for HheC wild type) while also exhibiting a 10 K higher apparent melting temperature. QM and MD simulations support the experimentally observed activity increase of HheC F12Y and reveal alterations in the hydrogen bonding network within the active site. As such, our results demonstrate that multiple enzyme properties of HHDHs can be altered through the targeted mutagenesis of conserved motif 1 residues. In addition, this work illustrates that motif 1 plays vital roles beyond nucleophile binding by impacting the solubility and stability properties. These insights advance our understanding of HHDH active sites and will facilitate their future engineering.

## Introduction

**Scheme 1:**
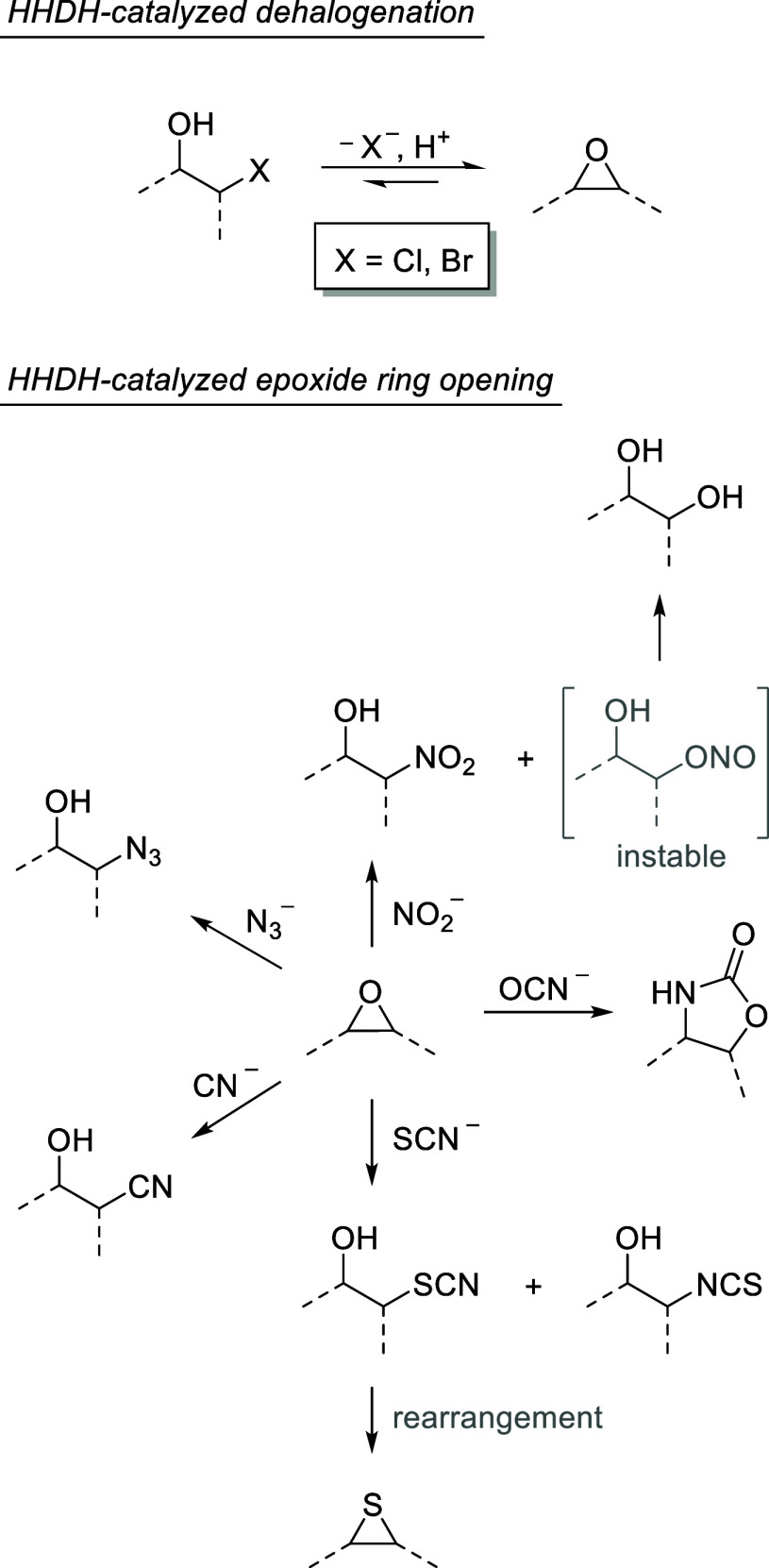
HHDH-Catalyzed Dehalogenation and Epoxide Ring Opening Reactions with Different *C*-, *N*-, *O*-, and *S*-Nucleophiles, Giving Access to a Large Repertoire of Valuable Products

Halohydrin dehalogenases (HHDHs) have recently distinguished themselves as powerful enzymes for the asymmetric diversification of oxy-functionalized synthons.^1−8^ In nature, these bacterial lyases catalyze the reversible dehalogenation of β-haloalcohols through formation of the corresponding epoxides.^9^ More importantly, they are capable of accepting a number of anionic *C*-, *N*-, *O*-, and *S*-nucleophiles in the reverse reaction, i.e., epoxide ring opening, giving access to a large repertoire of valuable products (Scheme 1).^10^ For instance, recent impressive biocatalytic examples for the application of HHDHs in asymmetric synthesis include the preparation of enantiopure β-nitroalcohols^11^ and thiiranes^12^ as well as the desymmetrization of 2-substituted-1,3-dichloro-2-propanols with subsequent cyanate-mediated ring opening to afford optically pure epoxides and oxazolidinones,^1^ among others.^7,13−15^ Moreover, the HHDH-catalyzed dehalogenation of γ- and δ-haloalcohols resulting in the formation of the corresponding oxetanes and tetrahydrofurans, respectively, as well as the ring opening of oxetanes, has been reported very recently.^16,17^

**Figure 1:**
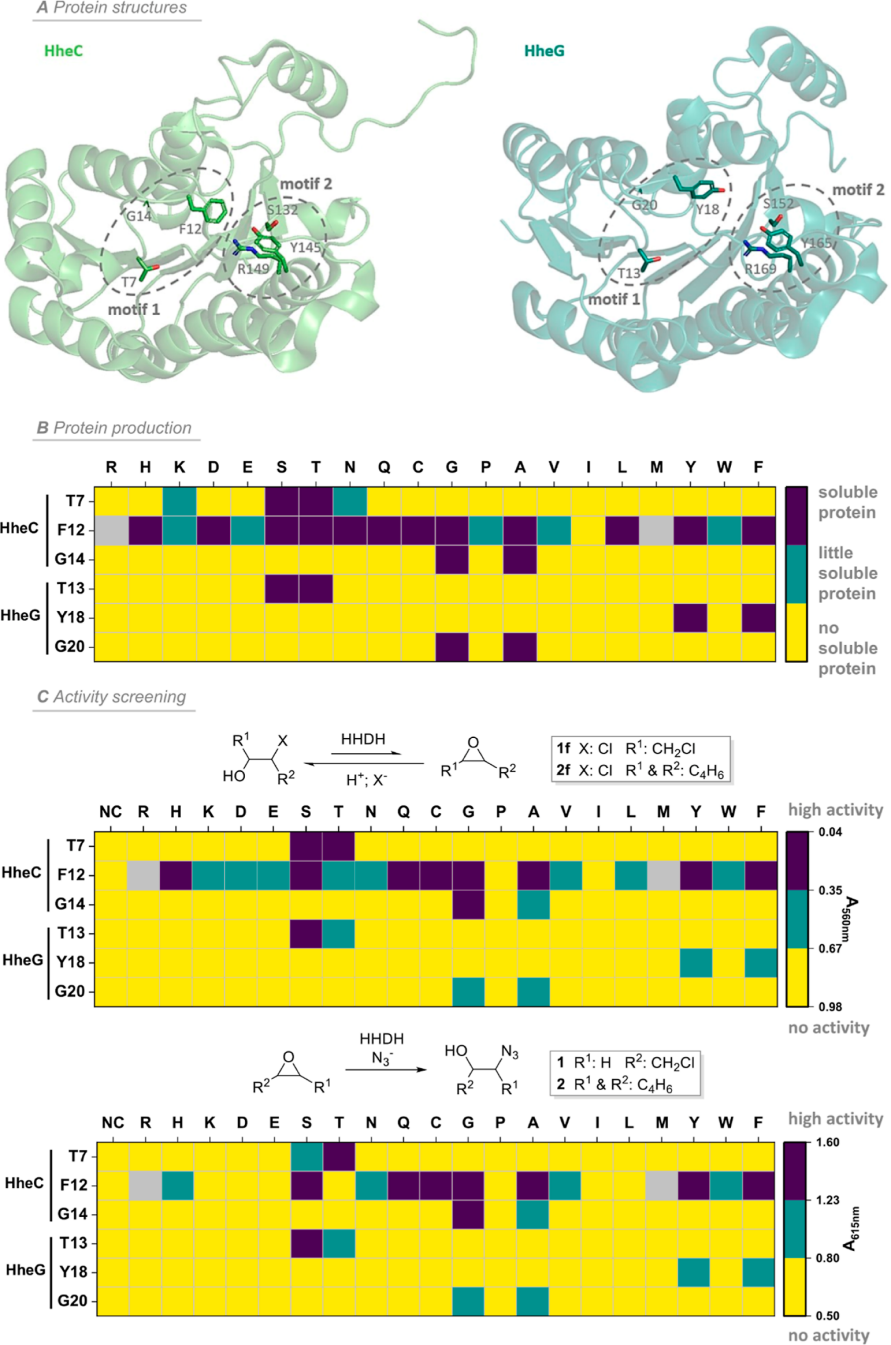
Summary of protein production and qualitative activity screening of motif 1 mutants of HheC and HheG. (A) Crystal structures of HheC (green; PDB: 1PWX) and HheG (blue; PDB: 5O30) WT with conserved residues of sequence motifs 1 and 2 highlighted. (B) Soluble expression of motif 1 mutants of HheC and HheG according to SDS–PAGE analysis (Figure S1). (C) Activity screening data (dehalogenation and epoxide ring opening) of motif 1 mutants of HheC and HheG using qualitative pH indicator-based assays. Substrates **1** and **1f** were used in combination with HheC, while the activity of HheG variants was analyzed toward substrates **2** and **2f**. High activity or solubility is represented by purple color, whereas no activity or no soluble protein is represented by yellow color. Gray color represents HheC mutants that could not be generated on the genetic level.

HHDHs share significant homology/similarity with short-chain dehydrogenases and reductases (SDRs) on the sequence, structural, and mechanistic levels as the result of a close phylogenetic relationship, although they catalyze entirely different chemical reactions.^18−20^ Previously, this similarity has largely impeded the fast discrimination of HHDHs and SDR enzymes solely based on given protein sequences. With the discovery of HHDH-specific sequence motifs in 2014, database mining approaches facilitated the identification of novel HHDHs and yielded a plethora of new enzymes with partially unprecedented catalytic characteristics.^21−23^ These HHDH sequence fingerprints include the N-terminal motif T-X_4_-F/Y-X-G (motif 1), lining the nucleophile binding pocket of HHDHs, as well as the motif S-X_12_-Y-X_3_-R (motif 2), covering the catalytic residues serine, tyrosine, and arginine (see Figure 1A).^18,20,24,25^ For comparison, the corresponding sequence motifs of SDRs are T-G/A-X_3_-G/A-X-G (motif 1)^19,21^ and S-X_10–14_-Y-X_3_-K (motif 2),^19,20^ respectively. In contrast to HHDHs, SDR motif 1 represents the typical glycine-rich nucleotide-binding sequence required for nicotinamide cofactor binding in those enzymes.^20^

While the mechanistic role of the three conserved catalytic residues serine, tyrosine, and arginine (as part of motif 2) of HHDHs has already been elucidated using HheC from *Agrobacterium radiobacter* AD1 as a model enzyme,^18,20,24,25^ the functional role and mutability of the conserved residues in motif 1 remain largely unexplored. Moreover, we hypothesized that HHDH variants with substantially improved biocatalytic performance could be accessed through the engineering of motif 1 residues, as they should impact nucleophile binding. Crystal structures of HheC in complex with a bromide or chloride ion (PDB IDs 1PWX and 1PWZ, respectively) have demonstrated that the side chain of the central aromatic residue F12 in motif 1 forms a direct interaction with the negatively charged halide, which is tightly wedged between aromatic residues.^20^ Such a direct interaction with the nucleophile, however, was not present in the crystal structure of HheB from *Corynebacterium* sp. strain N-1074 in complex with chloride (PDB ID 4ZD6), which carries a tyrosine at position 19.^26^ Moreover, in a study by Wu et al. on the thermostabilization of HheC by combinatorial directed evolution, mutation F12Y was found to increase the enzyme’s thermostability and yielded a 1.5-fold gain in specific activity in the dehalogenation of 1,3-dichloropropanol.^27^ Similarly, mutations at the structurally equivalent position Y15 in *Ab*HheG from *Acidimicrobiia* bacterium yielded variants with improved enantioselectivity in the ring opening of styrene oxide with cyanate quite recently.^14^ These examples directly hint at the hitherto underexplored possibility of steering the catalytic properties of HHDHs via targeted mutagenesis of sequence motif 1, which is the focus of this study.

In order to fully unveil the impact of sequence motif 1 on enzyme catalytic properties, we set out to systematically engineer the conserved motif 1 residues in two representative HHDHs, HheC from *A. radiobacter* and HheG from *Ilumatobacter coccineus*. We selected these HHDHs based on their biocatalytic relevance,^4,10,13,23,28−31^ the fact that both enzymes offer high-resolution crystal structures,^20,23,29^ as well as their highly dissimilar catalytic properties. HheC is by far the best-studied member of the HHDH family, generally displaying high catalytic activity and enantioselectivity in the dehalogenation of various substrates as well as in the ring opening of terminal epoxides.^3−5,18,32−36^ Moreover, numerous protein engineering studies of HheC have been published, aiming either at an altered substrate scope or an increased activity, enantioselectivity, and stability of the enzyme.^1,37−44^ The mechanism for the epoxide ring opening reaction in HheC has also been computationally investigated by Hopmann and Himo by means of quantum mechanics (QM) cluster model calculations,^24^ while the effect of motif 1 residues on the enzyme catalytic activity and conformational flexibility was not assessed. HheG, the second enzyme in this study, is rather thermolabile and much less selective. However, this HHDH was the first reported enzyme with relevant ring-opening activity toward sterically more demanding internal epoxides (cyclic as well as acyclic).^22,23,29−31,45^ Even though less protein engineering data are available for HheG so far, the enzyme exhibits distinct structural differences compared to HheC.^15,23,29,30,45−47^ This includes a much broader and open active site, an additional α-helix in the nucleophile binding site loop, as well as a long and highly flexible loop in the N-terminal part of the protein, modulating substrate access to but also substrate binding in the active site as well as enantioselectivity.^23,48,49^

In this work, we experimentally examined all possible single mutants of HheC and HheG with defined amino acid exchanges at the three conserved motif 1 residues, namely, threonine, phenylalanine/tyrosine, and glycine (Figure 1A), to investigate the impact of each residue on enzyme activity, selectivity, and stability. In this regard, our in-depth characterization revealed HheC F12Y to be enhanced considerably and toward multiple parameters, especially regarding epoxide ring-opening activity and enantioselectivity next to the previously observed thermostability increase.^27^ Complementary analysis of previously reported X-ray structures of HHDHs together with molecular dynamics (MD) and QM calculations highlighted the presence of an additional water molecule in the HheC wild type (WT), which is replaced by the hydroxyl group of Y12 in the F12Y variant. This additional hydroxyl group in mutant F12Y results in a better preorganization of the active site, retaining azide in the halide binding pocket and favoring its proper positioning toward the epoxide ring-opening reaction. In addition, a quantitative workflow for spectrophotometric activity determination in epoxide ring opening reactions has been developed, also enabling fast and reliable kinetic measurements.

## Results and Discussion

### Mutagenesis and Activity Screening

To study the impact of motif 1 mutations on the activity, selectivity, and stability of HHDHs, each of the three conserved residues of this motif (**T**-X_4_-**F/Y**-X-**G**) in HheC and HheG was individually replaced by the corresponding other 19 proteinogenic amino acids using either a Golden Gate-based or MEGAWHOP mutagenesis strategy.^50,51^ This resulted in a total of 112 defined single mutants, of which all could be generated successfully, except for mutants F12 M and F12R of HheC. Subsequent heterologous production of all 110 mutants in *Escherichia coli* BL21 (DE3) in 15 mL scale and partial purification via N-terminal His-tag using gravity flow revealed that only a few variants per position, usually carrying exchanges to structurally similar amino acids, still yielded observable amounts of soluble HHDH (Figures 1B and S1). Hence, this result suggests a possible direct impact of motif 1 residues on protein folding and stability. As an exception, position F12 of HheC permitted more diverse mutations, as almost all variants could be obtained as soluble proteins (Figures 1B and S1).

Next, all generated mutants were screened for their dehalogenation and epoxide ring-opening activity in a 96-well format. To this end, well-established model substrates for both the dehalogenation (dichloropropanol **1f** for HheC and chlorocyclohexanol **2f** for HheG) and the ring-opening reaction (epichlorohydrin **1** for HheC and cyclohexene oxide **2** for HheG) were used. For fast activity screening, we employed pH-based assays, which have previously been reported in the literature and make use of either phenol red or bromothymol blue as pH indicators to detect qualitatively the amount of released (dehalogenation) or consumed free protons (epoxide ring opening) during catalysis (Figure S2 and Table S2).^52,53^ For the exchange of the threonine (T7 in HheC and T13 in HheG) and glycine (G14 in HheC and G20 in HheG) in both enzymes, only mutants carrying a chemically similar amino acid (serine instead of threonine, alanine instead of glycine) still exhibited detectable activity. In contrast, F12 in HheC displayed a much higher variability (Figure 1C) with HheC mutants F12G, F12A, F12C, F12S, F12Q, F12H, and F12Y being active in both dehalogenation and epoxide ring opening reactions. Interestingly, this was not the case for position Y18 of HheG. Here, only the exchange of tyrosine with phenylalanine yielded a mutant with significant activity. Those activity data are in full agreement with our results regarding the soluble expression of the generated mutants, with the only exception being that not all soluble HheC F12X mutants were indeed also active. For all further tests, active HheC and HheG mutants of motif 1 were produced on a larger scale and applied as FPLC-purified proteins (for yields, see Table S1).

It should be noted here that Tian et al. do report a few active HheG mutants with amino acid exchanges at position Y18 of motif 1, which they obtained during protein engineering of this enzyme with the aim to improve enantioselectivity.^46^ In their case, however, only whole-cell reactions have been performed, while we have been working with isolated enzymes instead. Thus, it is possible that those HheG mutants exhibit even more reduced stability or yield much less soluble enzyme compared to wild-type HheG, which is why we could have lost them during enzyme isolation or purification in our study. At the same time, this would reinforce our assumption that position 18 in HheG impacts protein folding and stability.

To facilitate more detailed kinetic analyses of the epoxide ring-opening reactions catalyzed by HHDHs in high throughput, we developed a quantitative pH indicator assay inspired by work from Gul and colleagues.^52^ This assay relies on the conversion of a strong acid (e.g., azide) to a weak acid (e.g., an alcoholate) during epoxide ring opening, which increases the net pH value of the reaction mixture while the reaction progresses. Using bromothymol blue (**BTB**, an indicator with sweeping absorption spectra giving light yellow to dark blue mixtures) and dilute MOPS-buffered reaction mixtures, we typically followed ring-opening reactions starting at around pH 7. Unlike previously described assays using pH indicators,^52,53^ our system returns quantitative conversion data by employing isometric normalization.^54−56^ Using the isosbestic point of the deprotonation of **BTB**, we traced the deprotonation equilibrium of the indicator back to a concentration of consumed protons via the buffer strength (see the Supporting Information for details and mathematic operations). This assay proved readily compatible with high-throughput experimentation in 96-well plates and allowed straightforward monitoring of more than 20 reactions in parallel. Although we primarily used the assay for HHDH-catalyzed epoxide ring-opening reactions in our study, we expect it to be applicable to other proton-consuming or liberating (biocatalytic) reactions as well.

### Activity, Stability, and Enantioselectivity of Active Mutants

**Figure 2:**
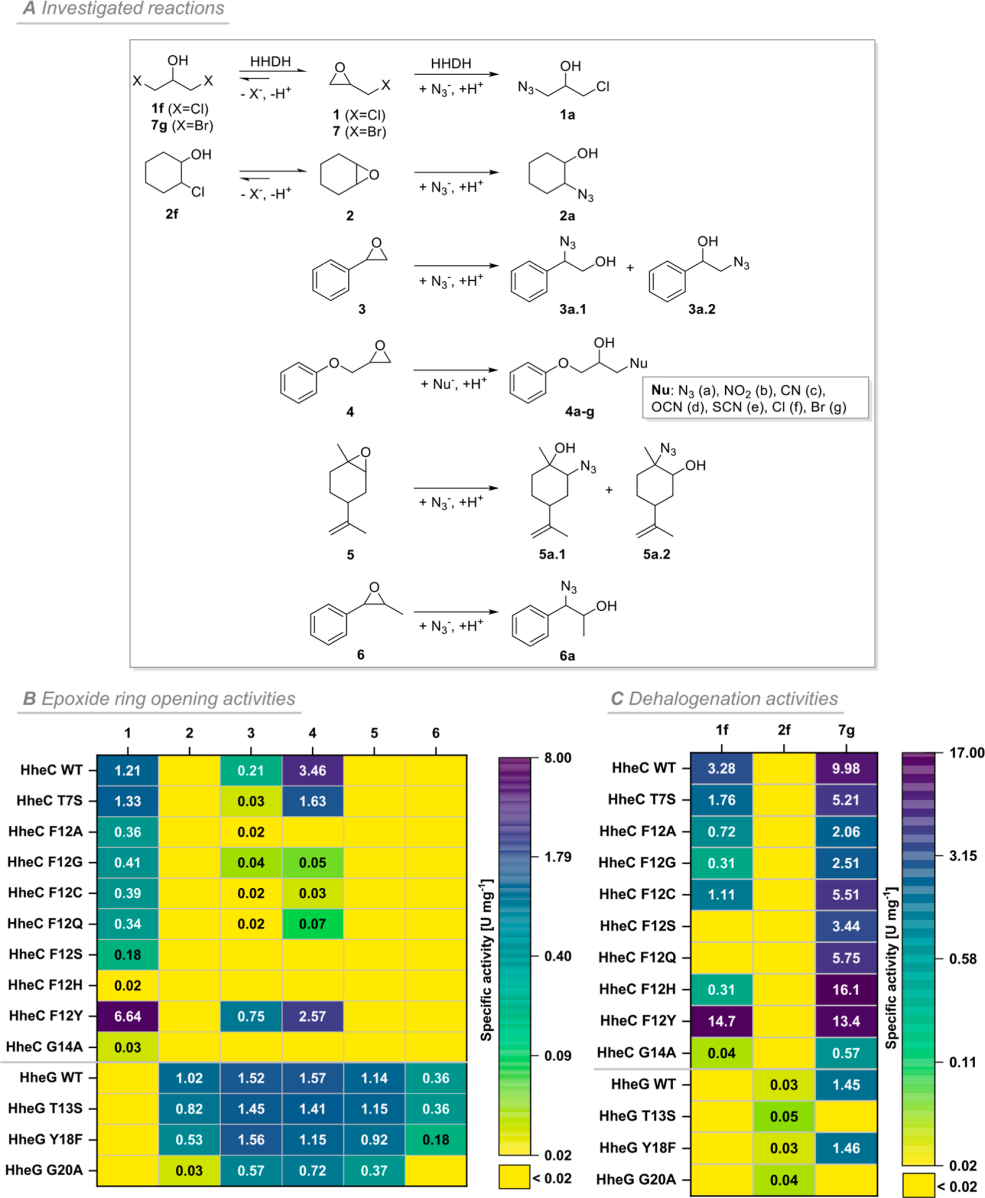
Specific activities (U mg^–1^) based on initial reaction rates of active motif 1 mutants of HheC and HheG in dehalogenation and epoxide ring opening reactions. (A) Overview of dehalogenation and epoxide ring opening reactions investigated in this study. (B) Specific activities of HheC and HheG mutants as well as wild-type enzymes in epoxide ring opening reactions of epichlorohydrin (**1**), cyclohexene oxide (**2**), styrene oxide (**3**), glycidyl phenyl ether (**4**), (+)-*cis/trans*-limonene oxide (**5**), and *trans*-1-phenylpropylene oxide (**6**) determined via the **BTB** assay. Reactions were performed in duplicate in a total volume of 1 mL with 10 mM epoxide and 20 mM azide in 2 mM MOPS buffer, pH 7.0 at 30 °C (HheC) or 22 °C (HheG) using 20–400 μg mL^–1^ purified enzyme (Table S3). Samples were taken after 30, 60, 180, 270, and 360 s. The chemical background of negative control reactions without enzyme addition was subtracted. The resulting specific activities exhibit standard deviations between 0.00 and 0.13 U mg^–1^. (C) Specific activities of HheC and HheG mutants as well as wild-type enzymes in the dehalogenation of haloalcohols 1,3-dichloro-2-propanol (**1f**), 2-chlorocyclohexanol (**2f**), and 1,3-dibromo-2-propanol (**7g**) determined via the halide release assay. Reactions were carried out in duplicate in a total volume of 1 mL with 10 mM haloalcohol in 25 mM Tris·SO_4_ buffer, pH 7.0 at 30 °C (HheC) or 22 °C (HheG) using 10–400 μg mL^–1^ purified enzyme (Table S4). Samples were taken after 30, 60, 180, 270, and 360 s. The chemical background of negative control reactions without enzyme addition was subtracted. The resulting specific activities exhibit standard deviations between 0.00 and 0.21 U mg^–1^.

Following the initial qualitative activity screen, we studied the epoxide ring-opening activity of active mutants with azide and various epoxides in more detail to gain further insight into the kinetic implications of motif 1 mutations on a broader range of substrates. Specifically, we used structurally diverse epoxides to cover relevant chemical space by employing epichlorohydrin (**1**), cyclohexene oxide (**2**), styrene oxide (**3**), glycidyl phenyl ether (**4**), limonene oxide (**5**), and *trans*-1-phenylpropylene oxide (**6**) (Figure 2A) and followed their conversion with our quantitative **BTB**-based pH assay. This analysis revealed that the introduction of mutations in motif 1 significantly impacted overall enzymatic activity (Figure 2B), even though the substrate spectra of the HheC and HheG mutants did not change compared with the WT. Indeed, most mutants exhibited considerable reductions of their specific activities with all model substrates compared to the respective wild-type enzyme. The only variant in this panel that gained significant activity was HheC mutant F12Y, displaying a 3- to 5-fold increase in specific activity with **1** and **3**. Otherwise, even very conservative mutations, as for instance the HheC mutant G14A and the HheG mutant G20A, resulted in drastic losses of activity. This observation is in general agreement with the report of Jörnvall et al. on the mutagenesis of the corresponding conserved glycine residue in motif 1 of an SDR enzyme. Replacement of this glycine by alanine resulted in a 69% decrease in activity, while mutations G14 V and G14N yielded almost inactive enzymes.^57^

The activity trends observed for the epoxide ring-opening direction generally carried over to dehalogenation catalyzed by the motif 1 mutants. We examined this with the haloalcohols 1,3-dichloro-2-propanol (**1f**), 2-chlorocyclohexanol (**2f**), and 1,3-dibromo-2-propanol (**7g**) (Figure 2A), whose conversion could be followed discontinuously with a previously reported halide release assay.^58^ While the specific activities of nearly all mutants were significantly reduced compared to those of the wild-type enzymes, HheC F12Y displayed considerably increased activity with the haloalcohols **1f** and **7g** (Figure 2C), mirroring the trends observed for the ring-opening activities. A higher activity of HheC mutant F12Y in the dehalogenation of 1,3-dichloro-2-propanol (**1f**) has previously been observed during the thermostabilization of HheC by directed evolution.^27^

To unveil if the observed activity increase of HheC mutant F12Y is induced by an improved substrate binding or rather a significantly elevated reaction rate, we measured kinetic data for the dehalogenation of haloalcohol **1f** and the ring opening of epoxide **1** with azide using the halide release assay^58^ as well as our **BTB**-based pH assay, respectively. Interestingly, this kinetic analysis revealed that the F12Y mutation only slightly impacted *K*_M_ or *K*_50_ for substrates **1f** and **1** (Table 1). In contrast, the maximal reaction rates were considerably increased, 3.5-fold for the dehalogenation of **1f** and at least 5-fold in the azidolysis of **1** (rate improvement varies when either the kinetics for epoxide or azide are considered). Thus, the improved performance of HheC F12Y in dehalogenation and epoxide ring opening is solely caused by an enhancement in reaction velocities. For comparison, the corresponding *K*_50_ values of HheC mutants T7S and G14A for binding of epoxide **1** were increased by a factor of 2 or more compared to the WT and mutant F12Y, indicating a lower substrate affinity of those motif 1 mutants in HheC. Moreover, HheC T7S exhibited an at least 2-fold higher *k*_obs,max_ compared to WT, while the respective *k*_obs,max_ of HheC G14A in the azidolysis of **1** was drastically reduced, both in line with our determined specific activities. Unfortunately, true *k*_cat_ values could not be determined in this epoxide ring opening reaction due to the high *K*_50_ values of all HheC mutants toward azide and the strong chemical background azidolysis of **1** occurring at azide concentrations above 100 mM. Therefore, kinetic measurements with varied epoxide concentration were performed at nonsaturating azide concentration, yielding significantly lower *k*_obs,max_ values compared to kinetic measurements with fixed epoxide and varying azide concentrations (Table 1). Importantly, all three HheC mutants do also exhibit higher *K*_50_ values as well as a considerably stronger cooperativity in azide binding—based on higher Hill coefficients *n*_H_—than the corresponding wild-type enzyme (Table 1). Thus, those conserved residues in sequence motif 1 of HheC indeed influence nucleophile binding to a great extent. In contrast, changes in the kinetic parameters of HheG mutants compared to WT in the azidolysis of epoxide **3** are less dramatic (Table 2), which is again in agreement with the respective specific activities of HheG mutants determined for this reaction. The observed cooperativity for azide binding, however, again varies significantly depending on the introduced mutation.

**Table 1:** Kinetic Parameters of Selected HheC Mutants in the Dehalogenation of 1,3-Dichloro-2-propanol (**1f**) (Determined by Halide Release Assay) as Well as the Azidolysis of Epichlorohydrin (**1**) (Determined by Our **BTB** Assay)^a^

			HheC			
	kinetic parameters		WT	T7S	F12Y	G14A
	*K*_M_	[mM]	0.45 ± 0.14	0.81 ± 0.23	0.98 ± 0.19	0.92 ± 0.37
**1f**	*k*_cat_	[s^–1^]	1.84 ± 0.11	1.37 ± 0.07	6.30 ± 0.26	0.05 ± 0.00
	*k*_cat_/*K*_M_	[mM ^–1^ s^–1^]	4.11 ± 1.33	1.70 ± 0.48	6.43 ± 1.33	0.06 ± 0.02
**1**	*K*_50_	[mM]	18.8 ± 1.24	40.3 ± 6.88	14.2 ± 0.67	49.5 ± 1.52
	*k*_obs,max_	[s^–1^]	8.16 ± 0.25	25.4 ± 1.90	60.2 ± 1.18	0.78 ± 0.03
	*k*_obs,max_/*K*_50_	[mM ^–1^ s^–1^]	0.44 ± 0.03	0.63 ± 0.12	4.24 ± 0.22	0.02 ± 0.00
	*n*_H_		1.70 ± 0.14	1.14 ± 0.10	2.32 ± 0.21	4.58 ± 0.79
**azide**	*K*_50_	[mM]	17.3 ± 2.29	80.9 ± 8.13	49.6 ± 2.49	111 ± 5.55
	*k*_obs,max_	[s^–1^]	27.0 ± 1.32	71.3 ± 4.72	137 ± 4.58	1.78 ± 0.08
	*k*_obs,max_/*K*_50_	[mM ^–1^ s^–1^]	1.55 ± 0.22	0.88 ± 0.11	2.76 ± 0.17	0.02 ± 0.00
	*n*_H_		1.61 ± 0.26	3.28 ± 0.64	2.44 ± 0.27	3.59 ± 0.57

a For the latter reaction, first the epoxide concentration was varied while keeping the azide concentration constant at 100 mM; afterwards the azide concentration was varied, fixing the epoxide concentration at 100 mM. The Michaelis–Menten equation was used to fit the resulting data for the dehalogenation of **1f**, whereas the Hill equation was used for fitting the experimental data obtained for the ring opening of **1** with azide.

**Table 2:** Kinetic Parameters of HheG Mutants in the Ring Opening of Cyclohexene Oxide (**2**) with Azide^a^

			HheG			
	kinetic parameters		WT	T13S	Y18F	G20A
**2**	*K*_50_	[mM]	39.4 ± 2.69	44.8 ± 1.29	66.4 ± 1.42	42.2 ± 3.64
	*k*_obs,max_	[s^–1^]	2.31 ± 0.15	3.34 ± 0.07	2.82 ± 0.06	1.02 ± 0.09
	*k*_obs,max_/*K*_50_	[mM ^–1^ s^–1^]	0.06 ± 0.01	0.07 ± 0.01	0.04 ± 0.00	0.02 ± 0.01
	*n*_H_		3.81 ± 0.78	3.67 ± 0.33	3.96 ± 0.25	3.28 ± 0.63
**azide**	*K*_50_	[mM]	38.4 ± 2.08	24.0 ± 1.59	26.4 ± 3.73	33.7 ± 0.57
	*k*_obs,max_	[s^–1^]	4.12 ± 0.15	5.31 ± 0.17	2.77 ± 0.17	1.74 ± 0.18
	*k*_obs,max_/*K*_50_	[mM ^–1^ s^–1^]	0.11 ± 0.01	0.22 ± 0.02	0.11 ± 0.02	0.05 ± 0.00
	*n*_H_		2.92 ± 0.36	2.40 ± 0.28	1.74 ± 0.27	3.90 ± 0.23

a The epoxide concentration was varied while keeping the azide concentration constant at 100 mM; afterward, the azide concentration was varied fixing the epoxide concentration at 100 mM. The Hill equation was used to fit the obtained experimental data.

Next, we probed the enantioselectivity of our motif 1 mutants in epoxide ring opening reactions with azide to test if motif 1 mutations also affected the selectivity of these enzymes. For variants of HheC, we selected the terminal epoxides **1** and **3** as substrates, while we used **3** and the cyclic epoxide **2** for HheG variants. Our choice of epoxide **3** was primarily motivated by the opposite regioselectivity of HheC and HheG in their ring opening of this substrate (Figure S3).^8^ The noncatalyzed reaction preferentially yields 2-azido-2-phenylethan-1-ol (**3a.1**) through nucleophilic attack at the benzylic α-carbon. While HheG enforces this inherent preference, HheC exhibits selectivity for attack at the terminal β-position. Biotransformations analyzed by chiral GC revealed that most of the motif 1 mutations in HheC decreased enantioselectivity significantly compared to the wild-type enzyme, independently of the epoxide substrate (Table 3). In contrast, mutants T7S and F12Y displayed a greatly increased enantioselectivity in the conversion of **1** with azide, while the extremely high enantioselectivity of the WT enzyme with **7** was maintained. This indicates a considerable impact of motif 1 residues on the enantioselectivity of HheC. On the other hand, the enantioselectivity of the studied HheG mutants in the ring opening of **2** and **3** hardly changed compared to HheG WT (Table 3). For comparison, mutations at the central aromatic residue Y18 in the homologous HheG enzyme from *Acidimicrobiia* bacterium did affect enantioselectivity in the ring opening of **3** with cyanate.^14^ Apart from this overall varying influence of motif 1 residues on enantioselectivity, all of the HheC and HheG mutants studied herein retained the wild-type enantiopreference for the conversion of (*S*)-**1** and (*R*)-**3**.

**Table 3:** Enantioselectivity of HheC Mutants and WT in the Azidolysis of 10 mM Epichlorohydrin (**1**) and Styrene Oxide (**3**), as Well as Enantioselectivity of HheG Mutants and WT in the Azidolysis of 10 mM Cyclohexene Oxide (**2**) and Styrene Oxide (**3**)^e^

HheC^a^									
	epoxide 1				epoxide 3				
mutant	enzyme conc. [μg mL^–1^]	*C* [%]	ee_P_ [%]	*E*	enzyme conc. [μg mL^–1^]	C_β_^b^ [%]	ee_S_ [%]	ee_Pβ_ [%]	*E*_β_^b^
NC		5.0	0.1			0.0^c^	0.0		
WT	10	46.5	82.6	22 (*S*)	25	29.1	35.4	99.9	>200 (*R*)
T7S	10	33.6	94.5	57 (*S*)	25	29.4	37.2	99.9	>200 (*R*)
F12A	400	50.9	62.5	8.3 (*S*)	400	28.4	72.8	36.7	2.5 (*R*)
F12G	400	51.1	45.5	4.2 (*S*)	400	19.5	73.2	39.1	2.5 (*R*)
F12S	400	20.8	46.2	3.1 (*S*)	400	20.4	73.2	25.9	1.8 (*R*)
F12C	400	40.4	64.5	7.0 (*S*)	400	16.9	0.83	45.3	2.9 (*R*)
F12Q	400	52.4	51.0	5.3 (*S*)	400	12.2	0.50	47.6	3.0 (*R*)
F12H	400	28.5	81.7	17 (*S*)	400	21.8	73.9	26.2	1.8 (*R*)
F12Y	10	45.3	93.8	74 (*S*)	25	34.5	44.7	99.9	>200 (*R*)
G14A	400	5.34	16.4	1.4 (*S*)	400	12.0	12.8	93.9	36.2 (*R*)

HheG^a^								
	epoxide 2			epoxide 3				
mutant	enzyme conc. [μg mL^–1^]	*C* [%]	ee_P_ [%]	enzyme conc. [μg mL^–1^]	*C*_α_^d^ [%]	ee_S_ [%]	ee_Pα_ [%]	*E*_α_^d^
NC		0.1	0.1		5.6	0.1	0.1	
WT	100	60.8	48.1 (*S*)	50	53.9	88.8	76.1	22 (*S*)
T13S	100	75.4	46.2 (*S*)	50	49.5	74.7	76.4	17 (*S*)
Y18F	100	53.6	56.1 (*S*)	50	48.6	72.4	76.5	16 (*S*)
G20A	400	23.6	50.6 (*S*)	400	43.3	32.1	42.2	3.3 (*S*)

a Reactions were carried out in a total volume of 1 mL in 50 mM Tris·SO_4_, pH 7.0, at 30 °C (HheC) or 22 °C (HheG) and 900 rpm using 10–400 μg mL^–1^ purified enzyme. Samples were taken after 15 min (epoxide **1**) or 1 h (epoxide **3**), extracted with an equal volume of *tert*-butyl methyl ether, and analyzed by achiral and chiral GC.

b Conversion and enantioselectivity toward the formation of product 2-azido-1-phenylethan-1-ol (**3a.2**) through nucleophilic attack at the terminal β-position.

c In the noncatalyzed chemical background reaction, the formation of product 2-azido-2-phenylethan-1-ol (**3a.1**) through nucleophilic attack at the benzylic α-carbon is preferred.

d Conversion and enantioselectivity toward the formation of product 2-azido-2-phenylethan-1-ol (**3a.1**) through nucleophilic attack at the benzylic α-carbon.

e “*C*” represents conversion, and “NC” represents negative control reactions without enzyme addition.

Following these activity and enantioselectivity studies, we also examined the thermal stability of active motif 1 mutants of HheC and HheG by differential scanning fluorimetry (also known as the thermofluor assay), as a positive impact of mutation F12Y on the thermostability of HheC has previously been reported.^27^ Our analysis revealed a considerable stabilizing effect for mutations F12H (+10.5 K), F12Y (+10.1 K), and G14A (+7.3 K) in HheC (Figure S4). The latter is especially surprising as this mutation heavily decreased enzyme activity. In contrast, the opposite trend in thermal stability has previously been reported for the exchange of the equivalent glycine residue in SDR motif 1 of *Drosophila* alcohol dehydrogenase^59^ and was also observed in our study for the corresponding mutation G20A in HheG. The herein reported stabilizing effect of mutation F12Y in HheC was previously attributed to the formation of an additional hydrogen bond with residue T131 compared to wild-type HheC, which we could confirm based on our computational results (see below).^27^ A slight increase (+2.8 K) in the apparent melting temperature of HheG upon exchange of the corresponding tyrosine 18 with phenylalanine becomes apparent from Figure S4 as well.

Taking into account that HheC mutant F12Y is not only more active in the dehalogenation and epoxide ring opening of several tested substrates but also exhibits higher enantioselectivity and thermal stability compared to wild-type HheC, the question arises why mutation F12Y was not selected during the natural evolution of this enzyme. For comparison, other native HHDHs such as HheB and HheG carry a tyrosine instead of a phenylalanine at the respective motif 1 position. One possible hypothesis might be that mutant F12Y is not superior in combination with HheC’s natural substrate(s), as the epoxides and haloalcohols tested by us probably do not represent natural substrates of HheC. In agreement with this hypothesis, mutant F12Y is not generally more active independent of the used substrate but was found to display a lower specific activity in the azidolysis of glycidyl phenyl ether. On the other hand, the gene of HheC is organized in an operon together with an epoxide hydrolase-encoding gene in the genome of *A. radiobacter*. Both enzymes were predicted to act together in the detoxification of harmful haloalcohols.^18^ Thus, HheC’s activity in *A. radiobacter* likely needs to be harmonized with the respective activity of the epoxide hydrolase to prevent an accumulation of the epoxide intermediate, which is harmful itself due to its high reactivity with, for example, primary amines of lysine residues. A too high activity of HheC might therefore not be evolutionarily beneficial.

### Nucleophile Acceptance of Active Motif 1 Mutants

**Figure 3:**
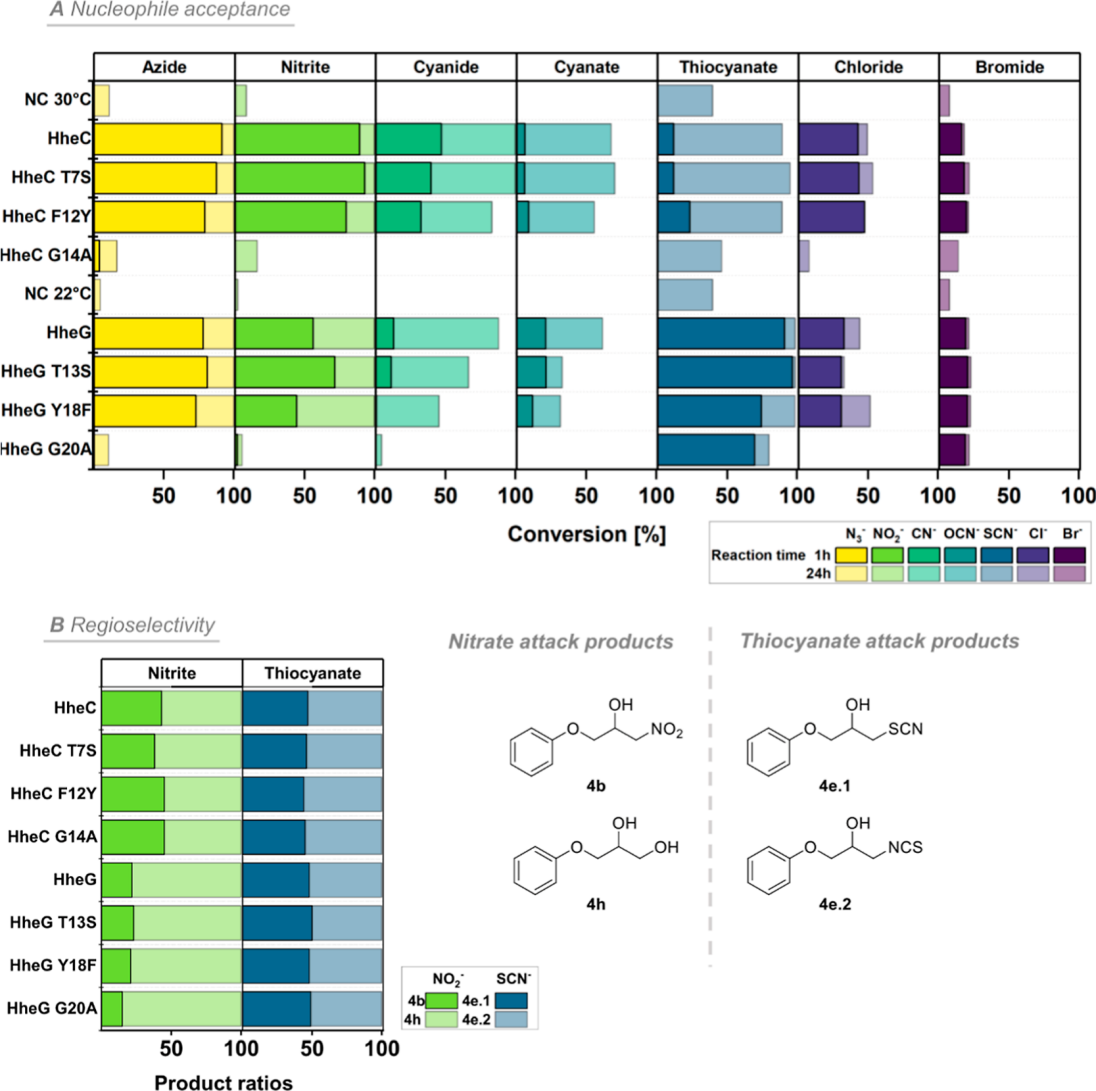
Nucleophile acceptance of motif 1 mutants. (A) Conversions of 10 mM glycidyl phenyl ether (**4**) with 20 mM nucleophile (azide, nitrite, cyanide, cyanate, thiocyanate, chloride, and bromide) in 50 mM Tris·SO_4_ buffer, pH 7.0, at 30 °C (HheC) or 22 °C (HheG) and 900 rpm using each 150 μg mL^–1^ purified enzyme. Reactions were carried out in a total volume of 1 mL. Samples were taken after 1 and 24 h, extracted with an equal volume of *tert*-butyl methyl ether and analyzed by achiral GC. “NC” represents negative control reactions without enzyme addition. (B) Ratio of formed products in reactions with nucleophiles nitrite and thiocyanate.

Since amino acids of sequence motif 1 line the nucleophile-binding pocket of HHDHs, we also expected a possible impact of those residues on nucleophile binding, as already observed for the nucleophile azide during our kinetic studies of motif 1 mutants. Thus, we further focused on a potential change in the nucleophile acceptance after mutagenesis of motif 1 residues using a broader range of nucleophiles. In this regard, mutants T7S, F12Y, and G14A of HheC as well as HheG mutants T13S, Y18F, and G20A were applied in epoxide ring opening reactions of glycidyl phenyl ether (**4**) using azide, nitrite, cyanide, cyanate, thiocyanate, as well as the halides chloride and bromide as nucleophiles. Those nucleophiles have previously been demonstrated to be accepted by HheC and HheG WT.^10,31^ To cover an activity range as large as possible, conversions of enzyme-catalyzed transformations were determined after short (1 h) but also extended (24 h) reaction times (Figure 3A). These experiments revealed that the overall nucleophile acceptivities of HheC and HheG were not altered considerably upon mutagenesis. However, a few interesting results stand out. For instance, HheG mutant G20A displayed surprisingly high activity with the nucleophiles thiocyanate and bromide, almost in the range of wild-type HheG, while it was virtually inactive with all other tested nucleophiles. However, the same effect was not noticed for HheC G14A, which might be related to the overall much lower activity of HheC with thiocyanate. As reported earlier,^31^ epoxide ring opening with thiocyanate can occur via *S*- and *N*-nucleophilic attack yielding two different product isomers, which were also observed in our study. Their ratio, however, did not change, depending on the applied enzyme variant (Figure 3B). In contrast, the ratio of formed diol and nitroalcohol product in the ring opening of **4** with nitrite as nucleophile indeed varied to some extent depending on the respective motif 1 mutation. In this case, the diol product occurs due to *O*-nucleophilic attack at the epoxide and subsequent hydrolysis of the formed nitrite ester. Interestingly, especially the preference of HheC mutant T7S and HheG mutant G20A for diol formation was increased in comparison to the respective wild-type enzymes (Figure 3B). Those results again underscore the impact of motif 1 residues on nucleophile binding and selectivity.

### Computational Analyses

**Figure 4:**
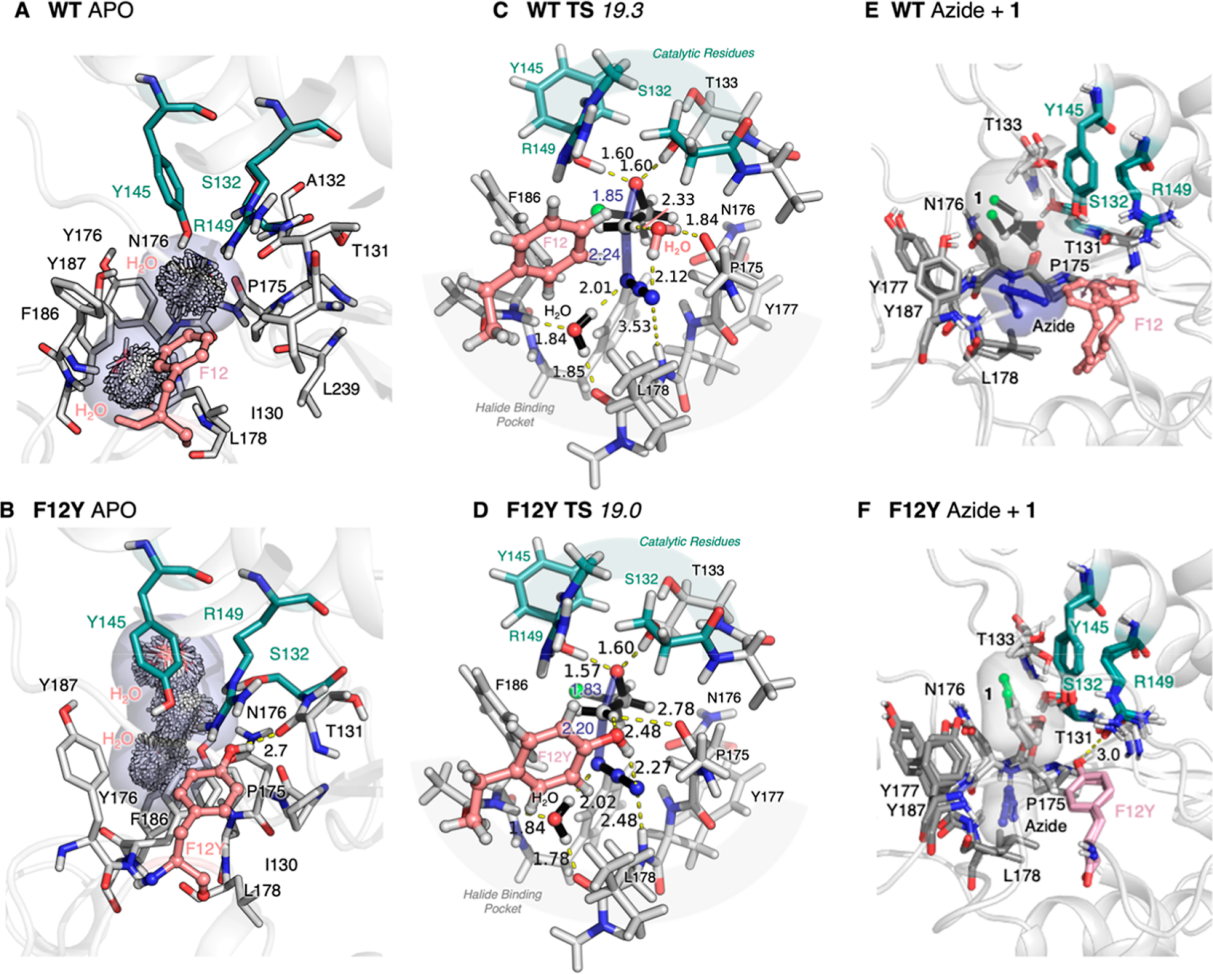
Computational evaluation of HheC WT and variant F12Y. Analysis of the water content of the active site for WT (**A**) and F12Y (**B**). QM-optimized TS for the epoxide ring-opening reaction of **1** with azide for: (**C**) **TS** for HheC WT and (**D**) variant F12Y. The computed activation energies are included in kcal/mol. Both **TSs** of WT, including a crystallographic water, and variant F12Y are very similar and present azide establishing hydrogen bonds with the backbone of L178 and the water molecule of the halide binding pocket. The nitrogen of azide undergoing the nucleophilic attack is hydrogen bonded to this water molecule, while the terminal nitrogen interacting with the backbone of L178 is either hydrogen bonded to the hydroxyl group of F12Y or the crystallographic water molecule in HheC WT. Overlay of two representative conformations extracted from the MD simulations performed with both epoxide **1** and azide for HheC WT (**E**) and variant F12Y (**F**). The extra flexibility of residue F12 in WT (highlighted with a double arrow) affects the productive binding of azide in the active site pocket, thus hampering the epoxide-ring opening reaction. All distances are in Å and energies in kcal/mol.

Intrigued by how the central aromatic residue in motif 1 enhances HheC activity toward the epoxide-ring opening reaction, we decided to computationally evaluate HheC WT and mutant F12Y by means of QM and MD simulations. Considering the proximity of position F12 to the catalytic residues (Figure 4A) and the big impact on the reaction rate (Table 1), we hypothesized that the additional hydroxyl group could potentially alter the water network of the active site and establish hydrogen bonds with either the catalytic and binding residues, the nucleophile binding pocket, and/or the substrates epichlorohydrin (**1**) and azide to promote catalysis. We first analyzed the water content of available X-ray structures of HHDHs grouped by whether they contain F or Y at position 12 (Figure S10). This comparison showed a higher water content in the case of F12-based X-ray structures, especially in the region close to P175, thus suggesting that in the WT of HheC a water molecule occupies the space of the additional hydroxyl group of Y12 in the variant. To characterize the active site water content and how this is altered by the F12Y mutation, we performed MD simulations of WT and variant F12Y in the absence of any substrate and analyzed the water content (Figure 4A,B). MD simulations confirmed a higher accumulation of water molecules in the active site pocket in the case of the WT, and in fact, one of the water clusters coincides with the observed X-ray water positioned close to P175. Based on this water analysis, we generated a cluster model as done previously by the group of Himo^24^ of the active site, including this crystallographic water molecule in the case of HheC WT, and used DFT to elucidate whether mutation F12Y directly impacts the activation barrier for the epoxide ring-opening reaction (Figures 4C,D and S11).^24^

We observed that both in the reactant complex (Figure S12) and the transition state (TS), the additional hydroxyl group, thanks to mutation F12Y, can establish a hydrogen bond with azide, which helps to retain the nucleophile in the nucleophile binding pocket and, more importantly, positions azide in a good orientation for epoxide-ring opening (Figure 4D). The terminal nitrogen of azide establishes a hydrogen bond with the backbone of L178 and the hydroxyl group of Y12, whereas the nitrogen involved in the nucleophilic attack is hydrogen-bonded to a crystallographic water molecule (Figure 4D). The hydroxyl group of Y12 also establishes a hydrogen bond with the hydrogen of the less substituted carbon of **1**. This interaction, together with the carbonyl group of P175, provides electrostatic stabilization to C_β_, thus favoring the attack at this position of the epoxide ring and enhancing the enantioselectivity of the reaction (Table 3).^24^ The activation barrier at this conformation toward the epoxide ring opening of **1** is *ca*. 19.0 kcal/mol at the M06-2X/Def2-TZVPP level of theory. This **TS** of variant F12Y is extremely similar to the one found for the HheC WT (Figure 4C), which in the place of the hydroxyl group of Y12 contains the additional crystallographic water molecule. This water is bridging the carbonyl backbone of P175 and the less substituted carbon of the epoxide substrate. The activation barrier for the ring opening of **1** in HheC WT is ca. 19.3 kcal/mol, i.e., only 0.3 kcal/mol higher in energy than in the case of F12Y. This small energy barrier difference does not, however, account for the large differences in the catalytic constants of WT and variant F12Y observed experimentally (minimum 5-fold increase in *k*_obs,max_).

We hypothesized that mutation F12Y could also help in the preorganization of the active site pocket, thus impacting and favoring the productive binding of the azide (and epoxide) in place for the ring-opening reaction to occur. To that end, we ran nanosecond time scale MD simulations for both HheC WT and mutant F12Y in the presence of epoxide **1** and azide in the active site. The analysis of the MD simulations in the absence and presence of both ligands indicated that F12Y establishes a hydrogen bond with the backbone of T131 that is adjacent to catalytic S132 (Figure 4B,F). This interaction established between the hydroxyl group of Y12 and T131, which is obviously not possible in HheC WT, has some important implications for the active site preorganization. Thanks to this interaction, the loop containing F12Y is slightly more rigid (Figure S13), which helps in retaining azide in place for the epoxide-ring opening reaction (Figure 4F). In the WT HheC, such an interaction is not possible; thus, the side chain of F12 is substantially more flexible and clearly affects the binding of the azide in the nucleophile binding pocket (Figure 4E). Moreover, this newly established hydrogen bond between Y12 and T131 and the resulting loop rigidification likely contribute to the observed higher thermal stability of HheC mutant F12Y as well, as suggested previously.^27^

Although the kinetic constant (*k*_obs,max_) for mutant F12Y is substantially improved, the mutation at the same time affects the binding of azide and induces a stronger cooperativity (Table 1). The higher *K*_50_ value found for azide in mutant F12Y can be explained by the distance between azide and position F12Y (Figure S14). In F12Y, azide can adopt two different binding modes: the catalytically productive pose, as shown in Figure 4D, and an additional one in which azide displaces the epoxide and interacts with both the catalytic S132 and F12Y (Figure S14). This additional, catalytically nonproductive binding mode likely causes the higher *K*_50_ value found experimentally for azide in mutant F12Y (Table 1). We additionally applied our correlation-based tool, the shortest path map (SPM),^60,61^ to investigate how the communication network between subunits might be altered through mutation F12Y (Figure S15). Interestingly, we observed a much more interconnected network in mutant F12Y, thus suggesting that the establishment of the Y12-T131 interaction enhances the intramolecular interactions and allosteric communication between subunits. This is in line with the higher Hill coefficient of HheC F12Y found experimentally (Table 1).

In summary, the QM and MD simulations of HheC WT and F12Y in complex with epoxide **1** and azide indicate that mutation F12Y changes the water content of the active site and the network of hydrogen bonding with azide, which has a small impact on the activation barrier (less than 0.5 kcal/mol) but clearly affects the preorganization of the active site and the retention of both epoxide and azide at their required optimal positions for enhanced activity. In comparison, the equivalent tyrosine 18 in the HheG WT is not able to establish similar hydrogen bonding interactions with azide or T151 (equivalent to T131 in HheC) due to the much wider active site pocket of HheG. The latter might explain why mutation Y18F in HheG affects enzyme activity only slightly (vide infra).

## Conclusions

Overall, we have demonstrated that important enzyme properties of HHDHs, such as activity, selectivity, and stability, are influenced by the conserved residues threonine, phenylalanine/tyrosine, and glycine of sequence motif 1 (**T**-X_4_-**F**/**Y**-X-**G**), which lines the nucleophile binding pocket of HHDHs. Despite the fact that those three residues appear to be highly conserved among naturally occurring HHDHs (please note that two highly homologous natural variants with sequence motif variation at the threonine position have been reported very recently^14,62^), we could show that especially the aromatic residue (phenylalanine/tyrosine) and the threonine position can be mutated to adjust enzyme properties, as exemplified for HheC. Only the conserved glycine residue of motif 1 proved quite invariable in both studied HHDHs, as even the exchange by alanine resulted already in drastic activity losses toward most tested dehalogenation and epoxide ring opening reactions.

Even though the actual effect of individual mutations is mainly enzyme-dependent, mutagenesis of motif 1 residues can yield greatly improved enzyme variants such as HheC F12Y. This mutant features not only a higher thermal stability as reported earlier^27^ but also displays much higher activity in the dehalogenation and epoxide ring opening of most substrates tested herein, as well as an impressive enantioselectivity improvement in the ring opening of epichlorohydrin (**1**), making this variant highly attractive for biocatalytic applications. Moreover, the detailed molecular insights into the activity improvement induced by mutation F12Y, which have been gained through QM and MD simulations in this study, will facilitate further protein engineering campaigns of HheC starting from mutant F12Y in the future.

## Experimental Section

### Chemicals

Substrates epichlorohydrin, 1,3-dichloropropanol, 1,3-dibromopropanol, and (*R*)-epichlorohydrin were purchased from Acros Organics (Geel, Belgium). Substrates cyclohexene oxide and styrene oxide were obtained from Thermo Fisher Scientific (Geel, Belgium). Substrates 2-chloro cyclohexane-1-ol, glycidyl phenyl ether, phenyl propylene oxide, limonene oxide, and (*S*)-epichlorohydrin were purchased from Merck (Darmstadt, Germany). Nucleophiles sodium azide, sodium nitrite, potassium chloride, potassium bromide, potassium cyanate, sodium cyanide, and sodium thiocyanate were purchased from Thermo Fisher Scientific (Geel, Belgium). (*R*)-Styrene oxide was purchased from abcr (Karlsruhe, Germany). All chemicals were of the highest available purity.

### Bacterial Strains and Plasmids

*E. coli* BL21(DE3) was used for heterologous protein production, as outlined before.^22,23,29^ All genes were expressed from vector pET-28a(+) (Merck) under control of the T7 promoter, resulting in the addition of an N-terminal hexahistidine (His_6_)-tag to the heterologously produced proteins.^21^

### HheC and HheG Mutagenesis

Amino acid positions for mutagenesis were selected based on the respective motif 1 sequences of HheC (T-X_4_-F-X-G) and HheG (T-X_4_-Y-X-G). For each conserved amino acid position within motif 1, variants carrying all 19 possible amino acid exchanges were generated using site-directed mutagenesis (see Table S6 for a list of used primers). In case of positions T13, Y18, and G20 of HheG as well as T7 and G14 of HheC, a Golden Gate mutagenesis protocol was used (see Supporting Information Table S7 for details regarding the composition of the PCR reaction).^50^ The PCR protocol for plasmid amplification while introducing the mutation consisted of an initial denaturation (98 °C, 30 s), 30 cycles of denaturation (98 °C, 10 s), annealing (*T*_m_-5 °C, 30 s), and elongation (72 °C, 30 s kb^–1^), followed by a final elongation step (72 °C, 120 s). After successful generation of mutated linear plasmids, one-pot restriction and ligation were performed using 1× cut smart buffer (NEB), 1× T4-ligase buffer, 2 U BsaI, 400 U T4-ligase, and 150 ng PCR product in 20 μL and was incubated for 2 h at 30 °C, followed by heat inactivation of the reaction for 20 min at 65 °C. Resulting plasmids were transformed into *E. coli* BL21(DE3) cells via the heat shock method.^63,64^ Correct insertion of the desired mutations was confirmed by sequencing.

For position F12 of HheC, a MEGAWHOP mutagenesis protocol was used.^51^ Respective MEGA primers were generated using QuikChange mutagenic reverse primers in combination with a T7 forward primer (Table S6). Reaction conditions (Table S7) as well as the PCR protocol were the same as for Golden Gate mutagenesis except for the used annealing condition, which was set to 57 °C for 30 s. For the subsequent MEGAWHOP mutagenesis, 300 ng of purified MEGA-primer, 30 ng of pET28a(+)-*hheC* and PfuUltra II Hotstart PCR Mastermix (Agilent Technologies, Santa-Clara, CA, United States) were applied. The PCR protocol consisted of an initial denaturation step (98 °C, 30 s), 30 cycles of denaturation (98 °C, 10 s), annealing (55 °C, 30 s), and elongation (68 °C, 2 min kb^–1^), followed by a final elongation step (72 °C, 120 s).

### Protein Production and Purification

Small-scale production of all HheC and HheG mutants was performed in 50 mL reaction tubes in a total volume of 15 mL of terrific broth (TB) (per liter: 4 mL of glycerol, 12 g of peptone, 24 g of yeast extract, 0.17 M KH_2_PO_4_, and 0.74 M K_2_HPO_4_) supplemented with 50 μg mL^–1^ of kanamycin and 0.2 mM isopropyl-β-d-thiogalactopyranoside and inoculated with 10% (v/v) preculture. After incubation for 24 h at 22 °C and 220 rpm, cells were harvested by centrifugation (20 min, 3488*g*, 4 °C), and the resulting cell pellets were stored at −20 °C until further use. For small-scale purification via N-terminal His-tag, cell pellets were resuspended in 2 mL of buffer A (50 mM Tris·SO_4_, 300 mM Na_2_SO_4_, and 25 mM imidazole, pH 7.9), supplemented with 1 mg mL^–1^ lysozyme and one Pierce Protease Inhibitor Mini Tablet (EDTA-free, Life Technologies, Thermo Fisher Scientific). Cells were disrupted by sonication on ice for 3 min (6 cycles of 10 s pulse and 20 s pause). Cell debris was removed by centrifugation (30 min, 21,000*g*, 4 °C). The resulting cell-free extracts were loaded on 0.8 mL Pierce centrifuge columns with a column volume (CV) of 0.6 mL Ni Sepharose 6 fast flow (GE Healthcare, Freiburg, Germany), pre-equilibrated with buffer A. After protein binding, columns were washed with each 10 CV of buffer A to remove nonspecifically bound proteins. Elution of His_6_-tagged target proteins was performed using 1.5 CV of buffer B (50 mM Tris·SO_4_, 300 mM Na_2_SO_4_, and 500 mM imidazole, pH 7.9), and fractions of each 1 mL were collected. For desalting, 1 mL of elution fraction was loaded onto PD MidiTrap G-25 desalting columns (GE Healthcare), pre-equilibrated with TE buffer (10 mM Tris·SO_4_, 4 mM EDTA, pH 7.9, 10% (v/v) glycerol), and eluted with 1.5 mL of TE buffer. Protein concentrations were determined based on absorbance at 280 nm using a NP80 nanophotometer (Implen, München, Germany) and respective molar extinction coefficients obtained by Protparam.^65^

Selected active mutants of HheC and HheG as well as wild-type enzymes were produced on a larger scale in shake flasks using the same protocol as mentioned above but 500 mL TB medium.^23^ Cells were harvested by centrifugation (20 min, 3494*g*, 4 °C), and the resulting cell pellets were stored at −20 °C until further use. For purification via N-terminal His-tag, cell pellets were resuspended in 30 mL of buffer A, supplemented with 1 mg mL^–1^ lysozyme and one Pierce Protease Inhibitor Mini Tablet, and disrupted by sonication on ice for 7 min (14 cycles of 10 s pulse and 20 s pause). Cell debris was removed by centrifugation (45 min, 18,000*g*, 4 °C), and the resulting cell-free extracts were filtered through a 0.45 μm syringe filter. Cell-free extracts were loaded (2 mL min^–1^ flow rate) on a 5 mL HisTrap FF column (GE Healthcare, Freiburg, Germany), pre-equilibrated with buffer A, using an ktaStart FPLC system (GE Healthcare). Afterward, the column was washed with 10 CV of buffer A to remove nonspecifically bound proteins. His_6_-tagged target protein was eluted using a gradient from 0 to 100% buffer B in 60 mL while collecting fractions of each 1 mL. Fractions with the highest UV absorbance at 280 nm were combined and concentrated to a volume of 2.5 mL using Vivaspin Turbo 15 centrifugation units (Sartorius, Göttingen, Germany) with a 10 kDa molecular weight cutoff. For desalting, the concentrated protein solutions were loaded onto PD10 desalting columns (GE Healthcare), pre-equilibrated with TE buffer, and eluted with 3.5 mL TE buffer. Respective yields of purified HheC and HheG variants are given in Table S1.

### pH-Indicator Assays for Initial Qualitative Activity Screening

All mutants of HheC and HheG were screened with regard to their dehalogenation and epoxide ring opening activities using two different pH-indicator assays. Dehalogenation reactions contained 2 mM HEPES·SO_4_ pH 8.2, 1 mM haloalcohol substrate [1,3-dichloropropanol (**1f**) or 2-chloro-1-cyclohexanol (**2f**) in case of HheC and HheG mutants, respectively], 20 μg mL^–1^ phenol red, and 50 μg mL^–1^ purified enzyme in a total volume of 200 μL.^53^ Reactions were performed in 96-well plates (Sarstedt, Nümbrecht, Germany) at 22 °C (HheG) or 30 °C (HheC) for 30 min with shaking at 500 rpm on an incubating microplate shaker (VWR, Darmstadt, Germany). Subsequently, the absorbance of each well at 560 nm was measured by using a ClarioStar microplate reader (BMG LABTECH GmbH, Ortenberg, Germany).

The epoxide ring opening activity was determined using bromothymol blue as the pH indicator.^52^ Reactions contained 10 mM epoxide substrates (cyclohexene oxide (**2**) or epichlorohydrin (**1**) in the case of HheG and HheC mutants, respectively), 20 mM azide, and 50 μg mL^–1^ purified enzyme in 2 mM MOPS·SO_4_ pH 7.0 in a total volume of 180 μL. Reactions were performed in 96-well plates (Sarstedt, Nümbrecht, Germany) at 22 °C (HheG) or 30 °C (HheC) for 30 min with shaking at 500 rpm on an incubating microplate shaker (VWR, Darmstadt, Germany). Subsequently, bromothymol blue was added to a final concentration of 20 μg mL^–1^ and absorbance of each well at 615 nm was measured using a ClarioStar microplate reader (BMG LABTECH GmbH, Ortenberg, Germany).

### Optimized Bromothymol Blue Assay for the Quantitative Analysis of Epoxide Ring Opening

For the determination of specific activities in epoxide ring opening with epoxide substrates **1**, **2**, **3**, **4**, **5**, and **6** using azide as a nucleophile, reactions of 1 mL total volume contained 10 mM epoxide, 20 mM azide, and 10–400 μg mL^–1^ purified enzyme in 2 mM MOPS·SO_4_ pH 7.0. Reactions were incubated at 22 °C (HheG WT and mutants) or 30 °C (HheC WT and mutants) while being shaken at 900 rpm in a ThermoMixer C from Eppendorf (Hamburg, Germany). Samples of each 100 μL were taken after 30–360 s and transferred to a 96-well plate containing already 100 μL quenching solution (40 μg mL^–1^ BTB in 100% methanol) per well. Afterward, absorbance at 616 and 499 nm was measured using a ClarioStar microplate reader. Activities were calculated using equations S8, S10, S15, S17, and S18 in the Supporting Information.

This assay was also used for the determination of kinetic parameters in epoxide ring-opening reactions. For kinetic measurement of HheC and its mutants, reactions were carried out in 2 mM MOPS·SO_4_ using 1–150 mM epichlorohydrin (**1**) while keeping the azide concentration fixed [60 mM for HheC WT, T7S, F12Y, and 100 mM for HheC G14A] or using 1–300 mM azide while keeping the concentration of epoxide **1** constant at 100 mM. Reactions were incubated at 30 °C. Samples were taken after 30–360 s to ensure determination of initial velocities. For HheG and its mutants, reactions were performed in 2 mM MOPS·SO_4_ using 1–150 mM cyclohexene oxide (**2**) while keeping the azide concentration fixed (60 mM for HheG WT, T13S, and Y18F, 100 mM for HheG G20A) or using 1–300 mM azide while keeping the concentration of epoxide **2** constant at 100 mM. Reactions were incubated at 22 °C. Samples were taken after 30–360 s to ensure determination of initial velocities. Analysis of the resulting kinetic data was performed as previously described for specific activity calculation, and kinetic data was fitted using the Hill-eq 1 in Origin Pro2021 (see Figures S6–S9).
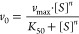
1

### Quantitative Analysis of Dehalogenation Activities

Specific activities (U mg^–1^) in the dehalogenation of haloalcohols 1,3-dichloro-2-propanol (**1f**), 2-chlorocyclohexanol (**2f**), and 1,3-dibromo-2-propanol (**7g**) based on initial reaction rates were determined using the halide release assay.^58^ Reactions were performed in duplicate in a total volume of 1 mL containing 10 mM haloalcohol in 25 mM Tris·SO_4_ buffer pH 7.0 at 30 °C (HheC and its mutants) or 22 °C (HheG and its mutants) using 10–400 μg mL^–1^ purified enzyme. Samples of 100 μL volume were taken after 30, 60, 180, 270, and 360 s and mixed with 100 μL of assay reagent comprising equal volumes of solution I [0.25 M NH_4_Fe(SO_4_)_2_ in 9 M HNO_3_] and solution II [saturated solution of Hg(SCN)_2_ in pure ethanol]. Absorbance at 460 nm was measured using a ClarioStar microplate reader. Specific activities were calculated using standard curves for halides Cl^–^ and Br^–^ in the range of 0 to 3.3 mM. The chemical background of negative control reactions without enzyme addition was always subtracted. This assay was also used for the determination of kinetic parameters in the dehalogenation of 1,3-dichloro-2-propanol (**1f**) by HheC mutants. For this, the same reaction conditions were used as described above with the substrate concentration ranging from 0.01 to 20 mM. Resulting kinetic data was fitted using the Michaelis–Menten eq 2 in Origin Pro2021 (see Figure S5).
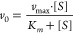
2

### Melting Temperature Determination

Thermal shift assays were performed using a QuantStudio 1 real-time PCR system (Thermo Fisher Scientific) in MicroAmp Optical reaction tubes (Thermo Fisher Scientific) containing 20 μg of protein and 10 μL of 50× concentrated SYPRO orange as fluorescent dye (Thermo Fisher Scientific) in TE buffer in a total volume of 50 μL. Fluorescence (excitation: 580 ± 10 nm, emission: 623 ± 14 nm) was monitored upon increasing the temperature from 10 to 90 °C in 0.5 °C increments. The temperature at which the maximum fluorescence change was observed, representing the melting temperature *T*_m_, was calculated using the Protein Thermal Shift software (version 1.4, Thermo Fisher Scientific).

### Determination of Nucleophile Acceptance

Mutants of HheC and HheG were analyzed in the epoxide ring opening of glycidyl phenyl ether (**4**) using azide, nitrite, cyanide, cyanate, thiocyanate, as well as chloride and bromide as nucleophiles. Each 1 mL reaction contained 10 mM epoxide **4**, 20 mM nucleophile, and 150 μg of purified enzyme in 50 mM Tris·SO_4_ buffer, pH 7.0. Reactions were carried out at 22 °C (HheG and its mutants) or 30 °C (HheC and its mutants) with shaking at 900 rpm in an Eppendorf ThermoMixer C. Samples were taken after 1 and 24 h, extracted with an equal volume of *tert*-butyl methyl ether containing 0.1% (v/v) *n*-dodecane as the internal standard. Organic phases were dried over MgSO_4_, and samples were analyzed by achiral GC (see Table S9 for details regarding temperature programs and retention times).

### Determination of Regio- and Enantioselectivity

To determine the regio- and enantioselectivity of HheC and HheG mutants in the epoxide ring opening of epichlorohydrin (**1**), cyclohexene oxide (**2**), and styrene oxide (**3**) in comparison to respective wild-type enzymes, reactions of 1 mL volume were performed in 50 mM Tris·SO_4_, pH 7.0, containing 10 mM epoxide, 20 mM azide, and 10–200 μg purified enzyme. Reactions were incubated at 22 °C (HheG and its mutants) or 30 °C (HheC and its mutants) with shaking at 900 rpm in an Eppendorf ThermoMixer C. Samples were taken after 15 min for epoxide **1**, 30 min for epoxide **3**, and 2 h for epoxide **2** and extracted with an equal volume of *tert*-butyl methyl ether containing 0.1% (v/v) *n*-dodecane as the internal standard. Organic phases were dried over MgSO_4_, and samples were analyzed by achiral and chiral GC (see Table S9 for details regarding temperature programs and retention times).

### MD Simulations

Parameters for substrates **1** and azide were generated with the antechamber and parmchk2 modules of AMBER20^66^ using the second generation of the general amber force field (GAFF2).^67,68^ Partial charges (RESP model)^69^ were set to fit the electrostatic potential generated at the HF/6-31G(d) level of theory. The charges were calculated according to the Merz–Singh–Kollman^70,71^ scheme using the Gaussian16 software package.^72^ The protonation states were predicted using PROPKA.^73,74^ The enzyme structures were obtained from the PDB with the code (1PWZ)^20^ and cleaned from other nonpeptidic molecules to obtain the WT system in a tetrameric oligomerization state. The single mutation F12Y was introduced by using the Pymol mutagenesis tool. Proteins were solvated in a pre-equilibrated truncated octahedral box of 12 Å edge distance using the OPC water model, resulting in the addition of ca. 21.300 water molecules, and neutralized by the addition of explicit counterions (i.e., Na^+^) using the AMBER20 leap module. All MD simulations were performed using the amber19 force field (ff19SB)^75^ in our in-house GPU cluster, GALATEA.

The Pmemd.cuda program from Amber20 was used to perform a two-stage geometry optimization. In the first stage, solvent molecules and ions were minimized, while solute molecules were restrained using 500 kcal·mol^–1^·Å^–2^ harmonic positional restraints. In the second stage, an unrestrained minimization was performed. The systems were then gradually heated by increasing the temperature by 50 K during six 20 ps sequential MD simulations (0–300 K) under a constant volume. Harmonic restraints of 10 kcal·mol^–1^·Å^–2^ were applied to the solute, and the Langevin equilibration scheme was used to control and equalize the temperature. The time step was kept at one fs during the heating stages to allow potential inhomogeneities to self-adjust. Each system was then equilibrated without restraints for 2 ns at a constant pressure of 1 atm and a temperature of 300 K using a 2 fs time step in the isothermal–isobaric ensemble (*NPT*). After equilibration, five replicas of 250 ns were run for each system (i.e., 1.25 μs per system and 5 μs in total simulated time) in the canonical ensemble (*NVT*). MD simulations were analyzed by monomers to make it easier to study, multiplying the simulated time by four. All analysis was done using available Python libraries (pyemma,^76^ mdtraj,^77^ and mdanalysis^78^) in a jupyter lab environment. Water cluster analysis was performed using the hydration sites analysis function included in the SSTMap software.^79^

### QM Calculations

For the QM cluster model, the atom selection was done following the previous work of Himo’s group.^24^ The differences with respect to Himo’s model are we added residues 185–187 and 179, completed L178, and included all backbone atoms for residue 12 (the position that is mutated in variant F12Y), allowing for additional flexibility and ring rotation. All terminal carbons, carbon alpha, and the added hydrogens are fixed during the QM calculations. Geometry minimizations were performed using Gaussian16,^72^ using the hybrid density functional theory method B3LYP,^80,81^ the 6-31G+(d,p) basis set, and including D3 dispersion corrections.^82^ All energies were calculated by performing single-point calculations on the optimized B3LYP/6-31G+(d,p) geometries using the M06–2x^83^ functional and Def2TZVPP basis set [M06-2X/Def2TZVPP//B3LYP/6-31+G(d,p)]. Solvation effects were considered using the SMD solvation model, a variation of Truhlar’s and co-workers’ integral equation formalism variant (IEFPCM),^84^ using diethyl ether as solvent.

### SPM Calculations

The SPM analysis^61,85^ was performed using the MD simulations of HheC WT and variant F12Y. For SPM calculation, the MD simulations are used to compute the inter-residue mean distance and correlation matrices. A simplified graph is created using both matrices in which only the pairs of residues that show a mean distance of less than 6 Å along the MD simulation are connected through a line. The edge connecting both residues is weighted to the Pearson correlation value (*d*_*ij*_ = −log|*C*_*ij*_|). The residues with more correlated motions will be connected through a shorter line. The generated graph is further simplified to identify the shortest path lengths. Following this strategy, the residues whose lines in the graph are shorter (i.e., with more correlated movements) and thus play an important role in the conformational dynamics of an enzyme are detected. Finally, the generated SPM graph is drawn on the three-dimensional structure of the enzyme.

## Supporting Information Available

Additional materials and methods descriptions; detailed information on the optimization of the employed bromothymol blue assay; supporting figures and tables regarding computational analyses as well as performed experiments of the study; and product characterization details (PDF)
