# Supplementary material for: Engineering of Conserved Sequence Motif 1 Residues in Halohydrin Dehalogenase HheC Simultaneously Enhances Activity, Stability, and Enantioselectivity
Source: ACS Catal. 2025 Mar 13;15(7):5257–72. doi: 10.1021/acscatal.5c00819 (PMC11976700; doi:10.1021/acscatal.5c00819)
Supplement: Supplementary file 1 — cs5c00819_si_001.pdf [file cs5c00819_si_001.pdf]

Supplementary information

Engineering of conserved sequence motif 1 residues in halohydrin dehalogenase HheC simultaneously enhances activity, stability and enantioselectivity

Sophie Staar[a], Miquel Estévez-Gay[b], Felix Kaspar[a,c], Sílvia Osuna*[b,d], Anett Schallmey*[a,e,f]

[a] Sophie Staar, Dr. Felix Kaspar, Prof. Dr. Anett Schallmey
Institute for Biochemistry, Biotechnology and Bioinformatics, Technische Universität Braunschweig, Spielmannstr. 7, 38106 Braunschweig, Germany
[b] Dr. Miquel Estévez-Gay, Prof. Dr. Sílvia Osuna
Institut de Química Computacional i Catàlisi (IQCC), Departament de Química, Universitat de Girona, c/Maria Aurèlia Capmany 69, 17003 Girona, Catalonia, Spain
[c] Dr. Felix Kaspar
Chair of Bioprocess Engineering, Institute of Biotechnology, Faculty III Process Sciences
Technische Universität Berlin
Ackerstraße 76, 13355 Berlin, Germany
[d] Prof. Dr. Sílvia Osuna
ICREA, Passeig Lluís Companys 23, 08010 Barcelona, Catalonia, Spain
[e] Prof. Dr. Anett Schallmey
Braunschweig Integrated Center of Systems Biology (BRICS), Technische Universität Braunschweig, Rebenring 56, 38106 Braunschweig, Germany
[f] Prof. Dr. Anett Schallmey
Center of Pharmaceutical Engineering (PVZ), Technische Universität Braunschweig, Franz-Liszt-Str. 35a, 38106 Braunschweig, Germany

* **Correspondence**
Prof. Dr. Anett Schallmey, Institute for Biochemistry, Biotechnology and Bioinformatics, Technical University Braunschweig, Spielmannstr. 7, 38106 Braunschweig, Germany.
Email: a.schallmey@tu-braunschweig.de

Prof. Dr. Sílvia Osuna, Institut de Química Computacional i Catàlisi (IQCC), Departament de Química, Universitat de Girona, c/Maria Aurèlia Capmany 69, 17003 Girona, Catalonia, Spain
E-mail: silviaosu@gmail.com

Content

Author contributions 3

Mutant expression and purification 3

Screening results for HheC and HheG mutants in dehalogenation and epoxide ring opening reactions 4

Activity, stability and enantioselectivity of active mutants 6

Kinetic measurements for selected HheC and HheG mutants in dehalogenation and epoxide ring opening 8

Computational analyses 12

Experimental section 15

- ***Mutagenesis*** 15
- ***Optimization of the BTB-based pH assay for quantification of epoxide ring opening activity*** 18
- ***Gas chromatography (GC)*** 21
- ***Product characterization*** 22

References 25

Author contributions

Conceptualization, A.S.; Data curation, S.S., M.E. and F.K.; Formal analysis, S.S., M.E. and F.K.; Funding acquisition, A.S. and S.O.; Investigation, S.S., M.E. and F.K.; Methodology, S.S., M.E. and F.K.; Project administration, A.S.; Resources, A.S. and S.O.; Software, M.E. and S.O.; Supervision, A.S. and S.O.; Validation, -; Visualization, S.S. and M.E.; Writing—original draft, S.S., A.S., M.E. and S.O.; Writing—review & editing, all authors.

Specifically regarding the experiments, S.S. generated all mutants on the genetic level, produced and purified them, and performed all wet-lab enzyme characterizations. Moreover, S.S. performed the preparative biotransformations toward the products **4a**-**4h**, which F.K. purified and characterized. F.K. conceptualized the theoretical framework for the quantitative BTB assay and performed together with S.S. the initial wet-lab work toward its implementation. S.S. developed the high-throughput workflows for its implementation in a discontinuous format and carried out all following experiments using this assay. M.E. performed all computational analyses including QM and MD simulations.

Mutant expression and purification

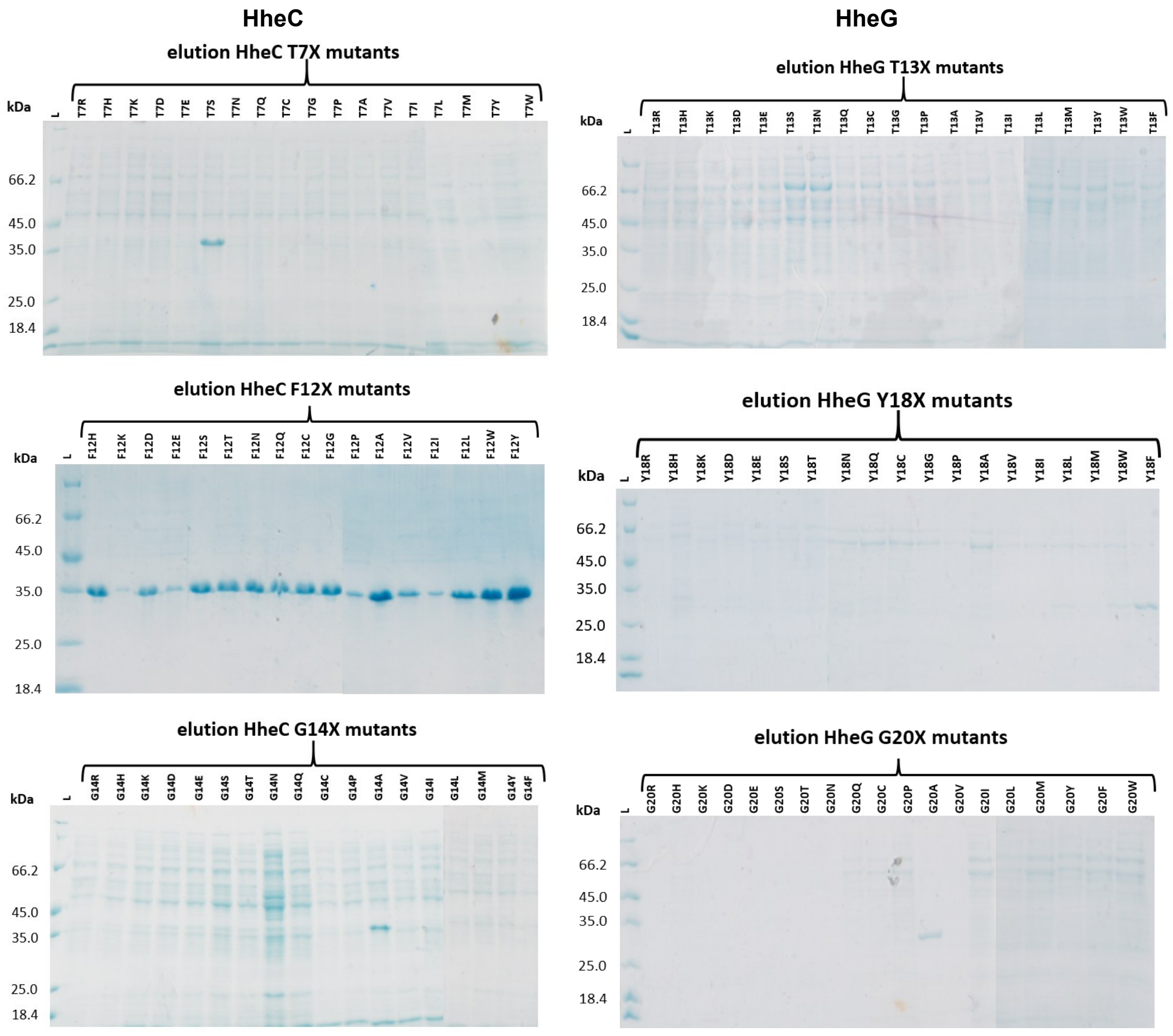

Figure S1. SDS PAGE analysis of elution fractions of HheC and HheG mutants after small-scale His-Trap purification. Molecular weights of HheC and HheG wild type are 28 kDa and 30 kDa, respectively.

Table S1. Yields after FPLC-based purification of HheC and HheG wild type (WT) and selected mutants from 500 mL expression cultures making use of the N-terminal His-tag.

Amino acid exchange	Yield [mg·L^{-1}$_{\text{expression volume}}$]					
	T7/13		F12/Y18		G14/20	
	HheC	HheG	HheC	HheG	HheC	HheG
WT	288	226	288	226	288	226
A	-	-	-	-	77.5	70.7
S	96.8	153	-	-	-	-
F	-	-	-	210	-	-
Y	-	-	232	-	-	-
H	-	-	307	-	-	-
A	-	-	443	-	-	-
G	-	-	354	-	-	-
S	-	-	296	-	-	-
C	-	-	240	-	-	-
Q	-	-	219	-	-	-

Screening results for HheC and HheG mutants in dehalogenation and epoxide ring opening reactions

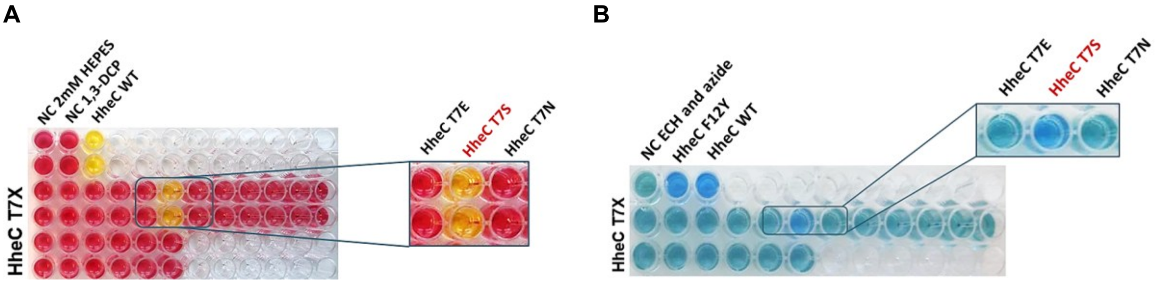

Figure S2. Exemplary pictures of pH-based screening reactions in 96-well plates after 30 min of incubation of selected HheC variants. **A.** Conversion of haloalcohol **1f** (10 mM) by HheC T7X mutants after 30 min in 2 mM HEPES·SO_4 buffer, pH 8.2 at 30°C. Inactive samples exhibit a red color like in the case of the negative control (NC) reaction without enzyme. Samples containing active enzyme are observed by their orange to yellow color, as in the case of HheC wild type (positive control) and variant T7S (highlighted in the box). **B.** Conversion of epoxide **1** (10 mM) by HheC T7X mutants after 30 min at 30°C in 2 mM MOPS·SO_4 buffer, pH 7.0 using azide (20 mM) as nucleophile. Here, inactive samples are visible by light-blue color, as observed for the negative control reaction without enzyme. Instead, samples containing active enzyme, such as HheC wild type, HheC F12Y and HheC T7S, exhibit a dark blue color.

Table S2. Summary of pH screening-based absorbance data in dehalogenation and epoxide ring opening reactions of HheC and HheG mutants. Dehalogenation activity was screened in conversions of 10 mM 1,3-dichloropropanol (**1f**) (HheC mutants) or 2-chlorocyclohexan-1-ol (**2f**) (HheG mutants) using the phenol red-based pH assay.[1] Activity in epoxide ring opening was screened in conversions of 10 mM epichlorohydrin (**1**) (HheC mutants) or cyclohexene oxide (**2**) (HheG mutants) with 20 mM azide using the bromothymol blue (BTB)-based pH assay.[2] Reactions were carried out in a total volume of 200 µL containing 2 mM HEPES buffer, pH 8.2 (dehalogenation) or 2 mM HEPES buffer, pH 7.0 (epoxide ring opening) for 30 min at 30 °C (HheC) or 22 °C (HheG) and 900 rpm. In case of dehalogenation, activity is represented by a decrease in absorbance. In contrast, activity in epoxide ring opening is represented by an increase in absorbance.

HheC

HheC	Dehalogenation screening (5), Absorbance 560nm	Ring opening screening (1), Absorbance 615nm
G14W	0.9405	0.562
G14F	0.987	0.562
G14Y	0.9365	0.567
G14M	0.955	0.555
G14L	0.9405	0.562
G14I	0.965	0.56
G14V	0.9495	0.57
G14A	0.8535	0.663
G14P	0.926	0.542
G14C	0.6555	0.51
G14Q	0.9045	0.52
G14N	0.946	0.521
G14T	0.9175	0.539
G14S	0.7895	0.487
G14E	0.8345	0.516
G14D	0.9215	0.546
G14K	0.8835	0.547
G14H	0.837	0.504
G14R	0.735	0.513
F12W	0.5355	0.8905
F12Y	0.0495	1.3805
F12L	0.6615	0.6095
F12I	0.6675	0.7195
F12V	0.558	0.8775
F12A	0.079	1.261
F12P	0.7005	0.6805
F12G	0.094	1.3155
F12C	0.099	1.37
F12Q	0.26	1.3555
F12N	0.4465	1.161
F12T	0.5875	0.635
F12S	0.162	1.3805
F12E	0.589	0.747
F12D	0.552	0.6575
F12K	0.643	0.774
F12H	0.181	1.2275
T7F	0.7925	0.617
T7W	0.9295	0.601
T7Y	0.7925	0.561
T7M	0.818	0.585
T7L	0.9855	0.618
T7I	0.8635	0.639
T7V	0.866	0.573
T7A	0.8075	0.612
T7P	0.787	0.617
T7G	0.8175	0.619
T7C	0.727	0.593
T7Q	0.8485	0.593
T7N	0.7995	0.588
T7S	0.066	0.88
T7E	0.8365	0.613
T7D	0.818	0.612
T7K	0.8335	0.64
T7H	0.9395	0.612
T7R	0.8775	0.639
HheC WT	0.0495	1.3805
NC	0.721	0.534

Absorbance 560nm scale (HheC dehalogenation): 1.00, 0.56, 0.32, 0.18, 0.10, 0.06, 0.03, 0.02, 0.01
Absorbance 615nm scale (HheC ring opening): 1.38, 1.16, 0.98, 0.82, 0.69, 0.58, 0.49

HheG

HheG	Dehalogenation screening (6), Absorbance 560nm	Ring opening screening (3), Absorbance 615nm
G20W	0.8938	0.528
G20F	0.8935	0.553
G20Y	0.9185	0.561
G20M	0.8375	0.572
G20L	0.913	0.564
G20I	0.9535	0.54
G20V	0.8585	0.596
G20A	0.667	0.826
G20P	0.8565	0.576
G20C	0.795	0.727
G20Q	0.805	0.58
G20N	0.9005	0.599
G20T	0.8445	0.632
G20S	0.9075	0.605
G20E	0.8435	0.603
G20D	0.898	0.716
G20K	0.823	0.577
G20H	0.8825	0.688
G20R	0.8435	0.625
Y18F	0.579	0.953
T18W	0.8935	0.5305
Y18M	0.9185	0.472
Y18L	0.8375	0.6355
Y18I	0.913	0.5305
Y18V	0.9535	0.525
Y18A	0.8585	0.5455
Y18P	0.867	0.548
Y18G	0.8565	0.506
Y18C	0.795	0.535
Y18Q	0.805	0.488
Y18N	0.9005	0.6245
Y18T	0.8445	0.554
Y18S	0.9075	0.5685
Y18E	0.8435	0.547
Y18D	0.898	0.5495
Y18K	0.823	0.6125
Y18H	0.8825	0.5375
Y18R	0.8435	0.552
T13F	0.825	0.5388
T13W	0.812	0.5065
T13Y	0.817	0.5775
T13M	0.807	0.562
T13L	0.828	0.6465
T13I	0.788	0.579
T13V	0.786	0.5455
T13A	0.816	0.5975
T13P	0.812	0.559
T13G	0.829	0.6065
T13C	0.804	0.5275
T13Q	0.86	0.5635
T13N	0.822	0.6245
T13S	0.574	1.673
T13E	0.825	0.582
T13D	0.841	0.6095
T13K	0.849	0.5685
T13H	0.824	0.5735
T13R	0.839	0.582
HheG WT	0.579	0.982
NC	0.838	0.534

Absorbance 560nm scale (HheG dehalogenation): 0.95, 0.90, 0.84, 0.79, 0.74, 0.69, 0.65, 0.61, 0.57
Absorbance 615nm scale (HheG ring opening): 1.68, 1.43, 1.22, 1.04, 0.89, 0.76, 0.65, 0.55, 0.47

Activity, stability and enantioselectivity of active mutants

Table S3. Enzyme amounts used for specific activity determinations in epoxide ring opening reactions of epoxide substrates **1**−**6** via quantitative BTB-assay.

**Amino acid exchange**	**Enzyme amount [µg]**					
	**T7/13**		**F12/Y18**		**G14/20**	
	**HheC**	**HheG**	**HheC**	**HheG**	**HheC**	**HheG**
-	100	100	100	100	100	100
A	-	-	-	-	400	200
S	100	100	-	-	-	-
F	-	-	-	100	-	-
Y	-	-	20	-	-	-
H	-	-	200	-	-	-
A	-	-	100	-	-	-
G	-	-	100	-	-	-
S	-	-	100	-	-	-
C	-	-	100	-	-	-
Q	-	-	100	-	-	-

Table S4. Enzyme amounts used for specific activity determinations in dehalogenation reactions via halide release assay. The used enzyme amounts are listed for the respective substrates given in brackets.

**Amino acid exchange**	**Enzyme amount [µg]**					
	**T7/13**		**F12/Y18**		**G14/20**	
	**HheC**	**HheG**	**HheC**	**HheG**	**HheC**	**HheG**
-	100 (**1f**)<br>400 (**2f**)<br>25 (**7g**)	400 (**1f**)<br>100 (**2f**, **7g**)	-	-	-	-
A	-	-	-	-	300 (**1f**)<br>400 (**2f**)<br>25 (**7g**)	400 (**1f**)<br>300 (**2f**, **7g**)
S	100 (**1f**)<br>400 (**2f**)<br>25 (**7g**)	400 (**1f**)<br>100 (**2f**, **7g**)	-	-	-	-
F	-	-	-	400 (**1f**)<br>100 (**2f**, **7g**)	-	-
Y	-	-	50 (**1f**)<br>400 (**2f**)<br>25 (**7g**)	-	-	-
H	-	-	100 (**1f**)<br>400 (**2f**)<br>25 (**7g**)	-	-	-
A	-	-	100 (**1f**, **7g**)<br>400 (**2f**)	-	-	-
G	-	-	100 (**1f**, **7g**)<br>400 (**2f**)	-	-	-
S	-	-	100 (**1f**, **7g**)<br>400 (**2f**)	-	-	-
C	-	-	100 (**1f**, **7g**)<br>400 (**2f**)	-	-	-
Q	-	-	100 (**1f**, **7g**)<br>400 (**2f**)	-	-	-

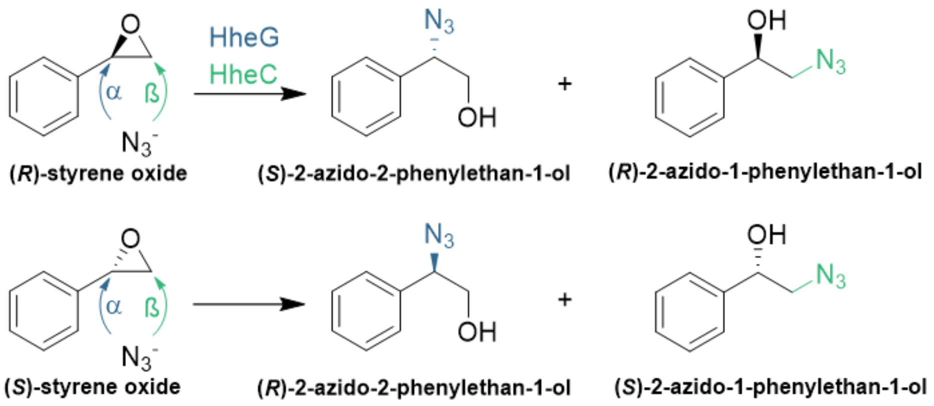

Figure S3. Opposite regioselectivity of HheC and HheG in ring opening reactions of styrene oxide (**3**).

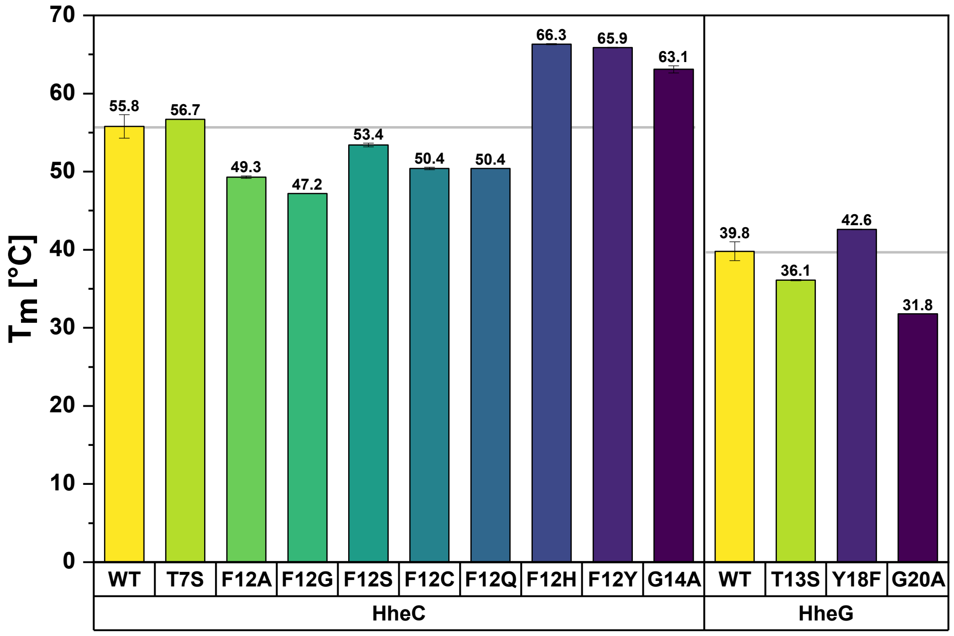

Figure S4. Apparent melting temperatures (T_m) of active HheC and HheG mutants as well as wild-type (WT) enzymes determined via thermofluor assay.

Kinetic measurements for selected HheC and HheG mutants in dehalogenation and epoxide ring opening

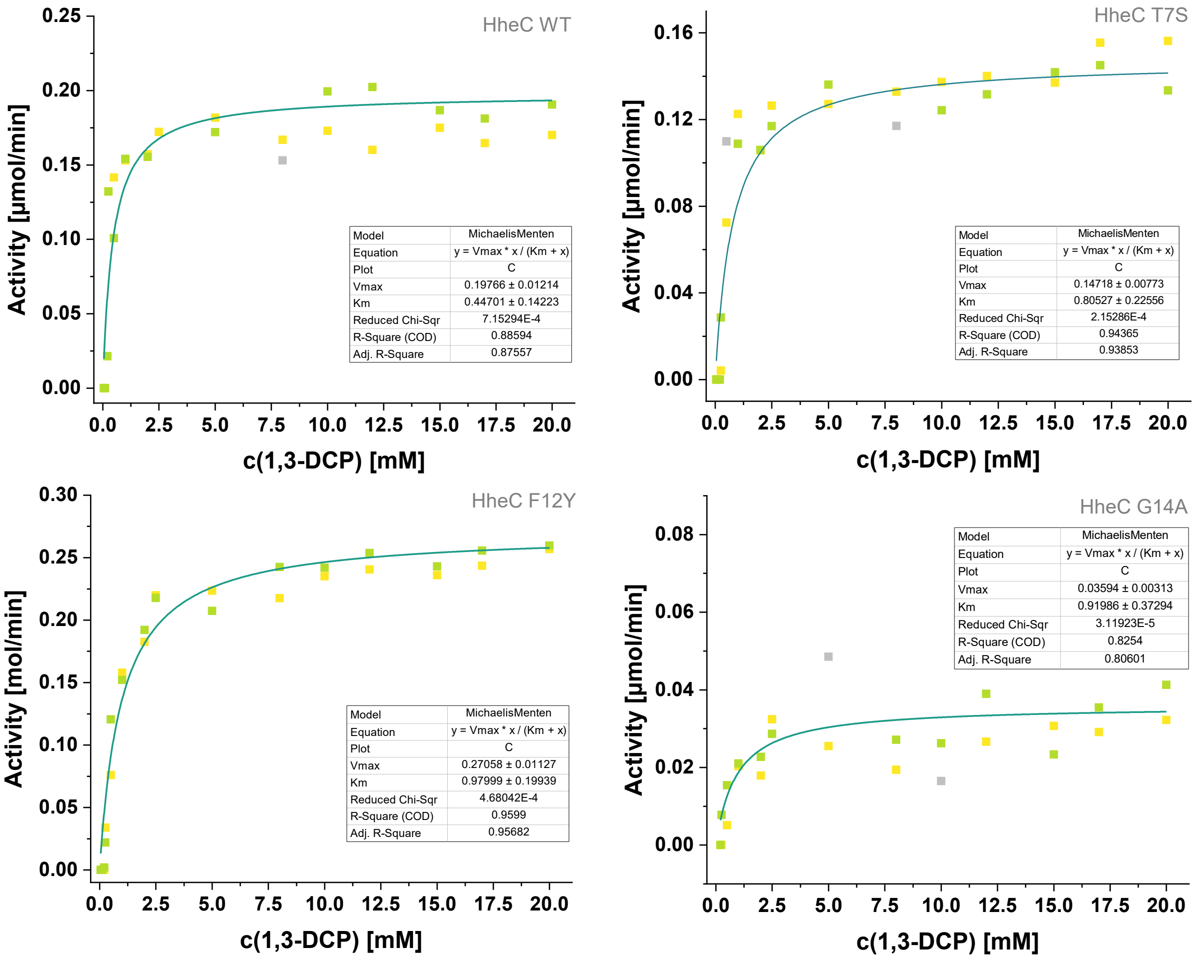

Figure S5. Kinetic measurements of HheC wild type and mutants T7S, F12Y and G14A in the dehalogenation of 1,3-dichloropropan-2-ol (1,3-DCP,**1f**) as determined by halide release assay. Reactions were caried out in 25 mM Tris·SO_4 buffer, pH 7.0 at 30 °C. Samples were taken in a time range of 30−360 s. Data from two independent measurements are shown in green and yellow. Resulting data were fitted to the Michaelis-Menten equation in Origin Pro.

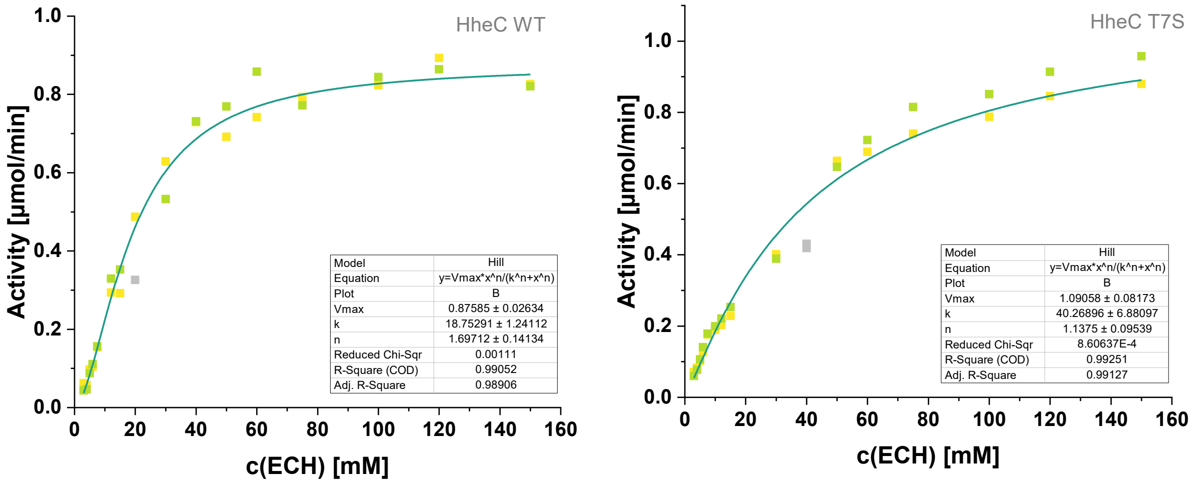

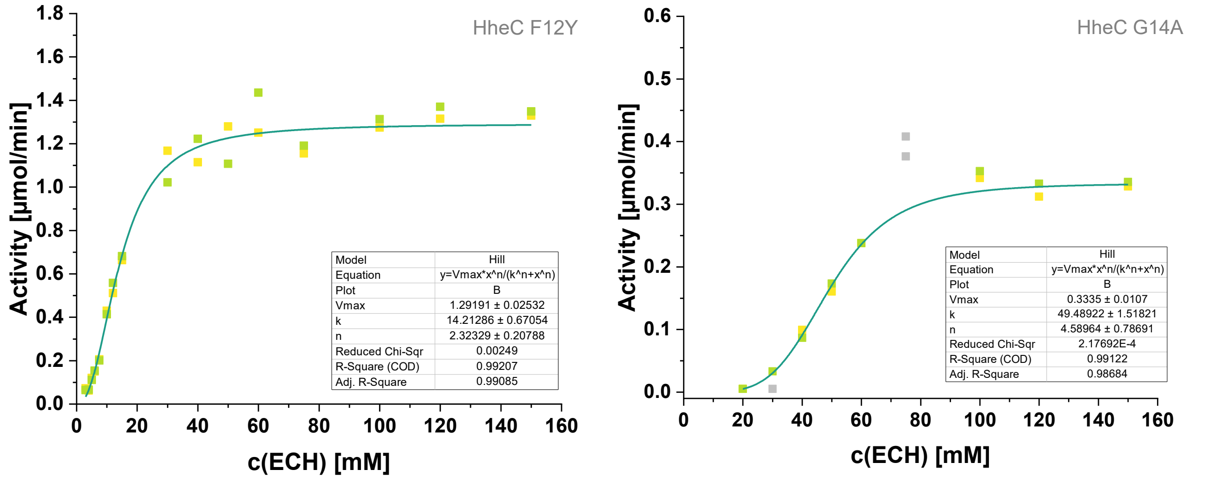

Figure S6. Kinetic measurements of HheC wild type and mutants T7S, F12Y and G14A in the ring opening of epichlorohydrin (ECH, **1**) with azide as determined by BTB assay. The epoxide concentration was varied while the azide concentration was fixed at 60 mM (or 100 mM for mutant G14A). Reactions were caried out in 2 mM MOPS·SO_4 buffer, pH 7.0 at 30 °C.Samples were taken in a time range of 30−360 s. Data from two independent measurements are shown in green and yellow. Resulting data were fitted to the Hill equation for cooperative substrate binding in Origin Pro.

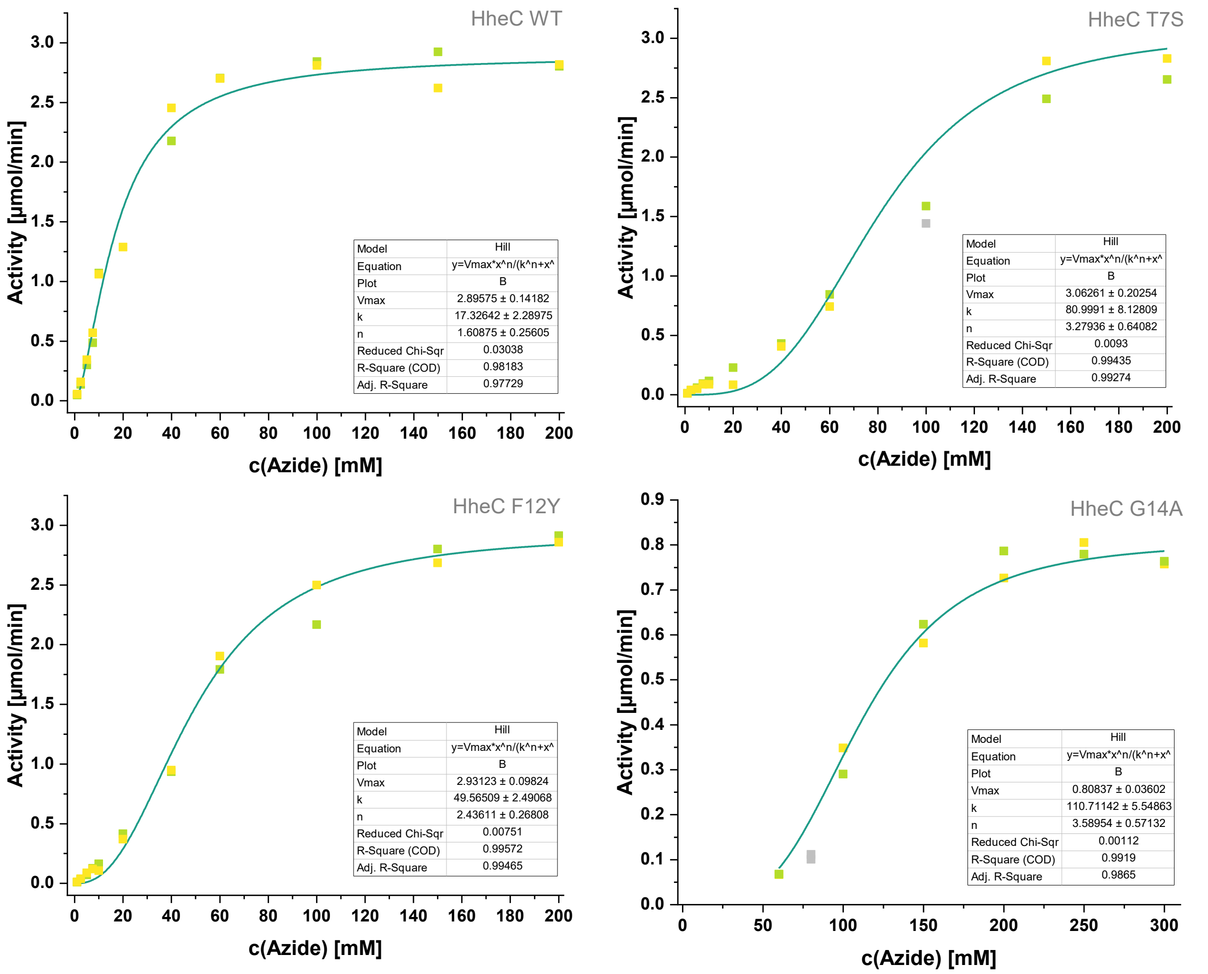

Figure S7. Kinetic measurements of HheC wild type and mutants T7S, F12Y and G14A in the ring opening of epichlorohydrin (**1**) with azide as determined by BTB assay. The concentration of the nucleophile azide was varied while the epoxide concentration was fixed at 100 mM. Reactions were caried out in 2 mM MOPS·SO_4 buffer, pH 7.0 at 30 °C. Samples were taken in a time range of 30−360 s. Data from two independent measurements are

shown in green and yellow. Resulting data were fitted to the Hill equation for cooperative substrate binding in Origin Pro.

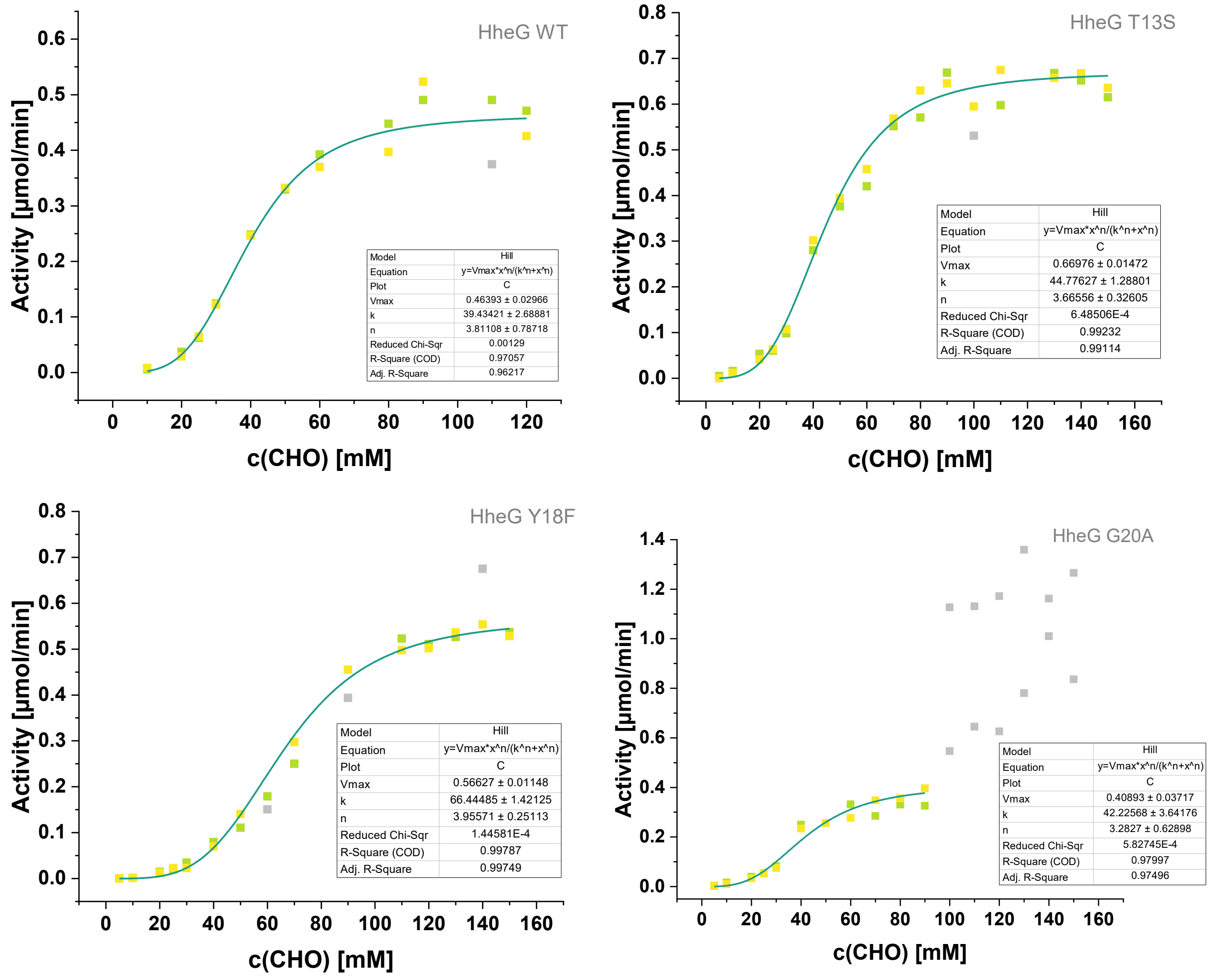

Figure S8. Kinetic measurements of HheG wild type and mutants T13S, Y18F and G20A in the ring opening of cyclohexene oxide (CHO, **2**) with azide as determined by BTB assay. The epoxide concentration was varied while the azide concentration was fixed at 60 mM (or 100 mM for mutant G20A). Reactions were caried out in 2 mM MOPS·SO_4 buffer, pH 7.0 at 22 °C. Samples were taken in a time range of 30−360 s. Data from two independent measurements are shown in green and yellow. Resulting data were fitted to the Hill equation for cooperative substrate binding in Origin Pro.

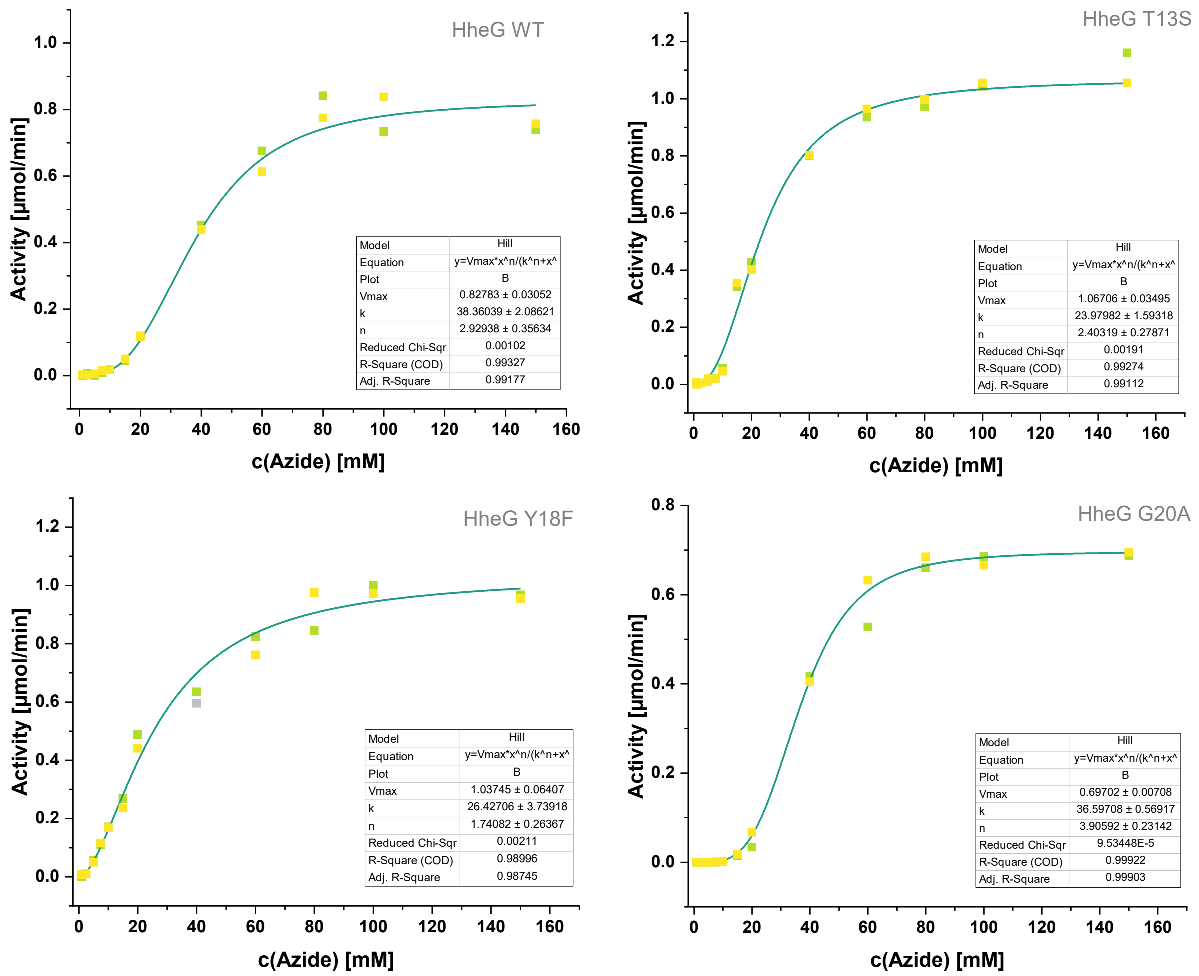

Figure S9. Kinetic measurements of HheG wild type and mutants T13S, Y18F and G20A in the ring opening of cyclohexene oxide (**2**) with azide as determined by BTB assay. The concentration of the nucleophile azide was varied while the epoxide concentration was fixed at 100 mM. Reactions were caried out in 2 mM MOPS·SO_4 buffer, pH 7.0 at 22 °C. Samples were taken in a time range of 30−360 s. Data from two independent measurements are shown in green and yellow. Resulting data were fitted to the Hill equation for cooperative substrate binding in Origin Pro.

Computational analyses

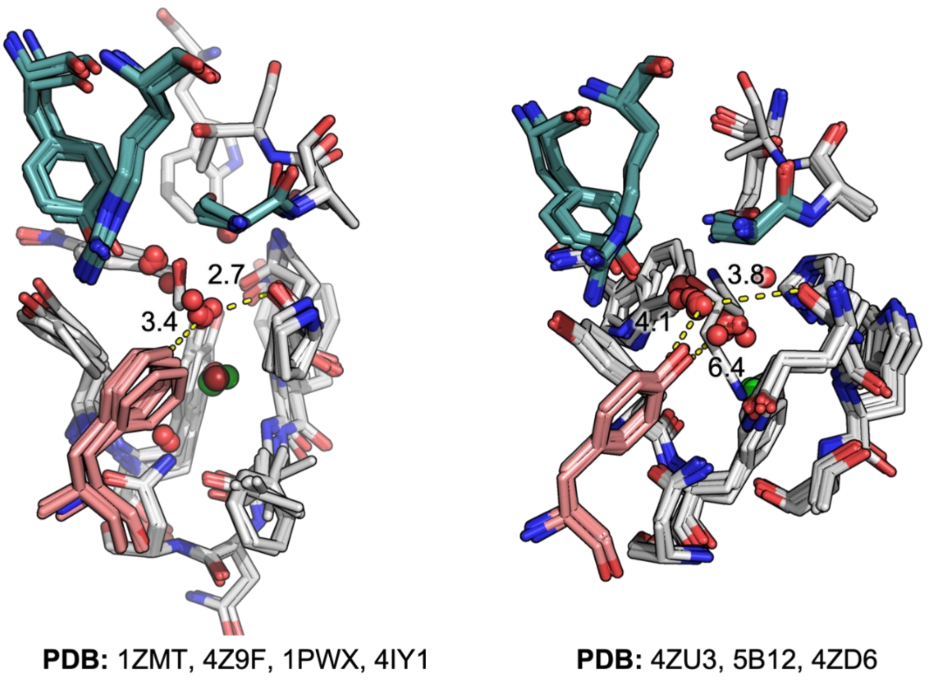

Figure S10. Analysis of the available X-ray structures of HHDHs. (Left) X-ray structures presenting a phenylalanine at the structurally equivalent position of F12 of HheC present some water molecules accumulated between F12 and P175 (HheC numbering). (Right) X-ray structures presenting a tyrosine at the structurally equivalent position of F12 of HheC contain crystallographic water molecules further away, mostly accumulated at the substrate binding positions. The PDB codes of used X-ray structures are indicated.

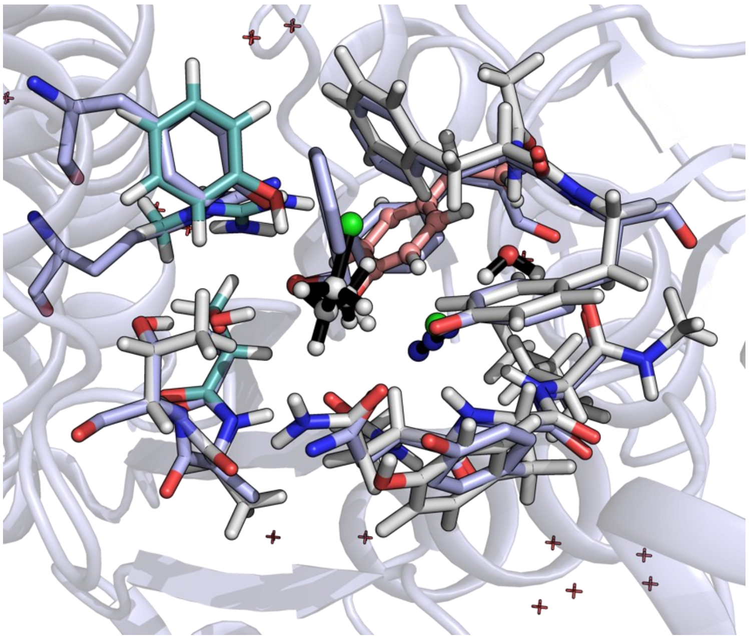

Figure S11. Overlay of the generated cluster model of variant F12Y with the X-ray structure of HheC wild type (PDB: 1PWZ). The cluster model is shown in gray, except for the catalytic residues shown in light teal, and position 12 that is shown in salmon. The X-ray structure of HheC wild type is shown in light blue.

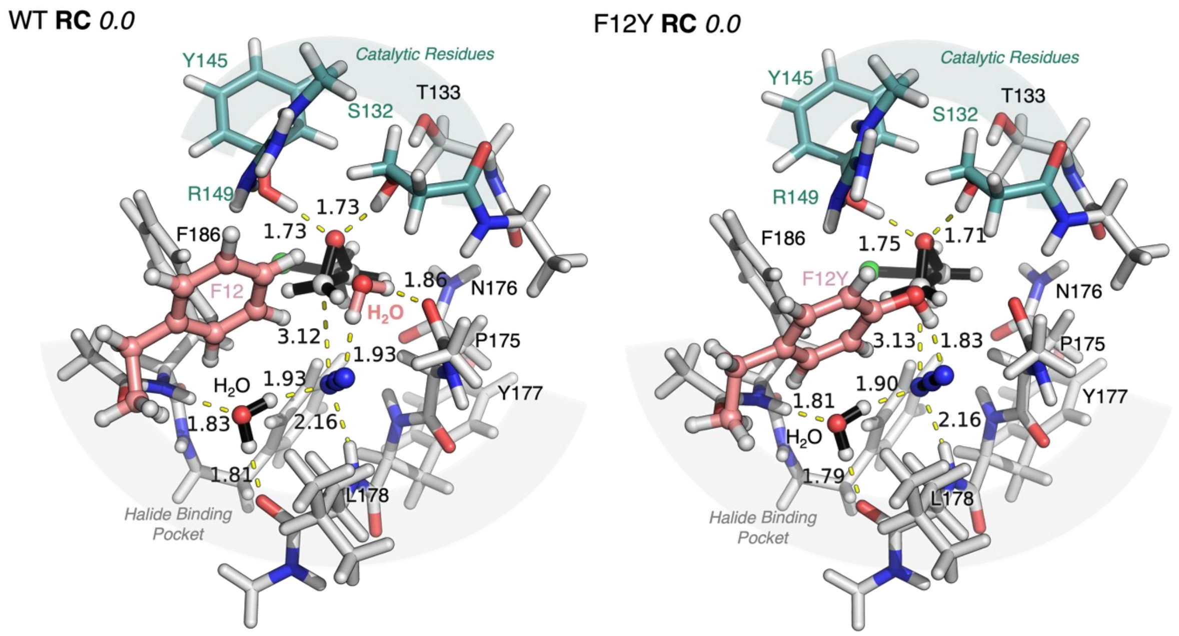

Figure S12. QM optimized Reactant Complexes (RC) for the epoxide ring-opening reaction of **1** with azide for HheC wild type (WT) and variant F12Y. All RC are very similar and present azide establishing hydrogen bonds with the backbone of L178 and a water molecule in the nucleophile binding pocket.

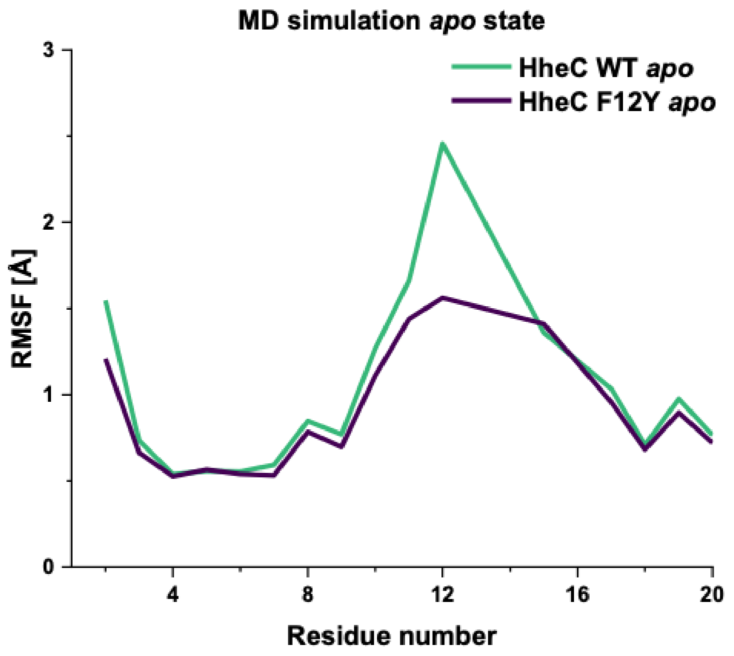

Figure S13. Root Mean Square Fluctuation (RMSF) computed considering the C_β atoms along the nanosecond timescale MD simulations in the absence of azide and epoxide. A slightly higher flexibility of residue 12 in HheC wild type is observed compared to variant F12Y.

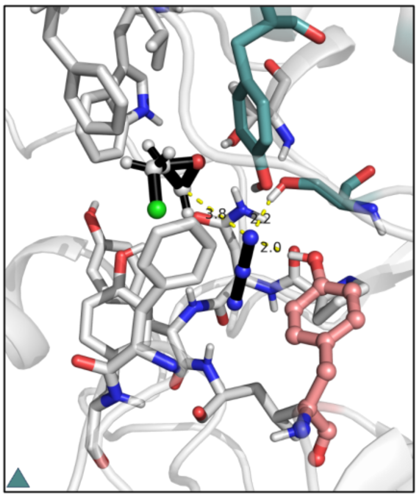

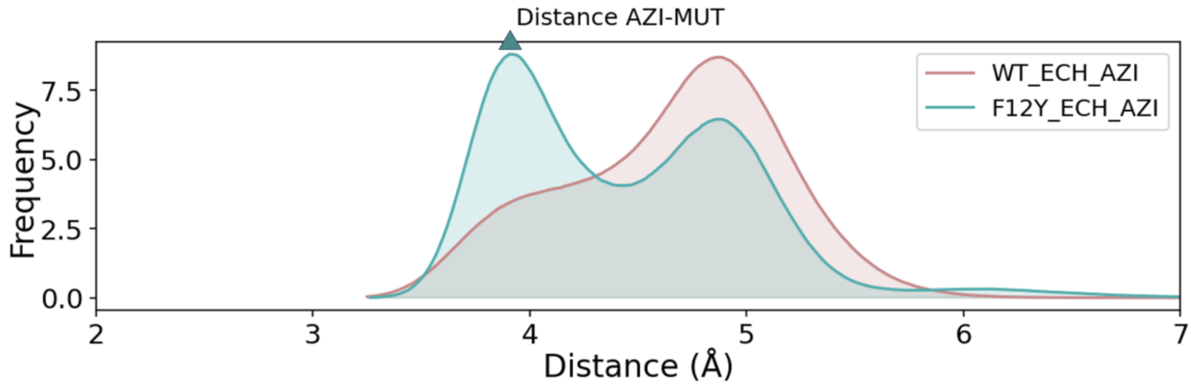

Figure S14. Histogram plot of the distance (in Å) between azide and the side-chain carbon atom of residue 12 in HheC presenting either a proton (wild type) or a hydroxyl group (variant F12Y). In red-brick color the data for the HheC wild type (WT) system is represented, whereas teal is used for the data of the HheC F12Y system [ECH = epichlorohydrin (**1**)**,** azi = azide]. A representative structure of the additional binding mode of azide. found mostly in the case of variant F12Y, is shown on top. The most relevant distances are given in Å.

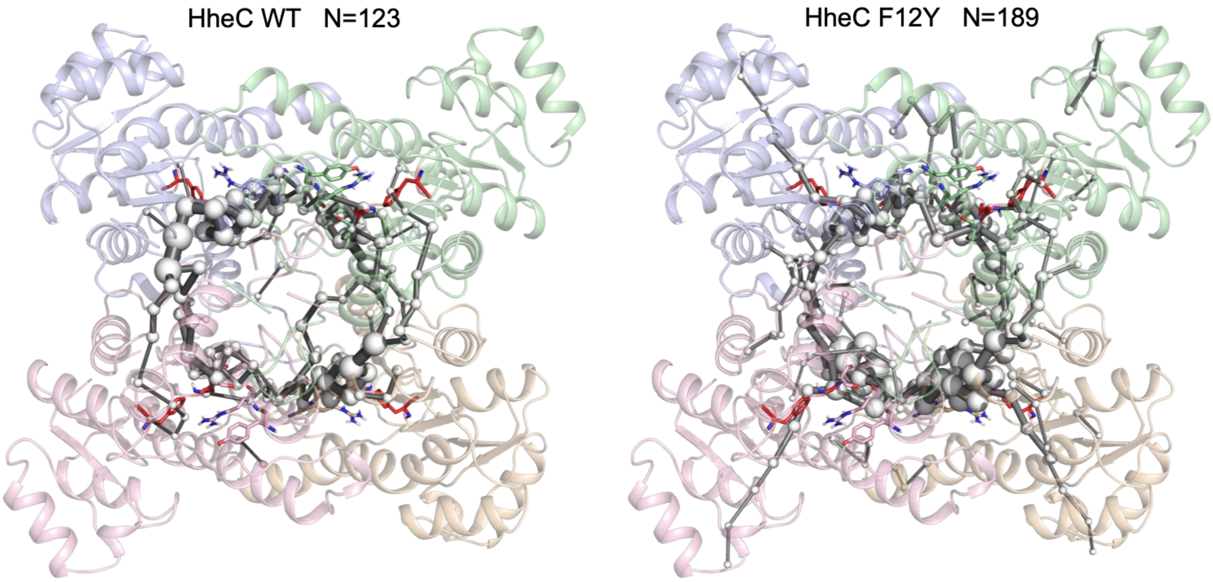

Figure S15. Cartoon representation of wild-type HheC (HheC WT, on the left) and HheC F12Y (on the right), depicting in colors the different monomers. In red sticks are shown for the residues F12/Y12 and T131. SPM graphs are shown in gray sticks and white spheres on top of the tetrameric structures. SPM is computed using a 6 Å

distance's threshold and a 0.2 significance's threshold, 123 residues are identified in WT system (N=123), whereas 189 residues in the F12Y variant. The establishment of the F12Y-T131 interaction enhances the intramolecular interactions and thus the allosteric communication between subunits (in line with the higher Hills coefficient found experimentally).

Experimental section

Mutagenesis

Table S6. Mutagenic primers used in this study. Nucleotide exchanges as well as the BsaI restriction site are highlighted with capital letters. MegaWhop primers are highlighted with capital letters as well.

Mutagenic primer	Sequence 5'-3'
Fw_HheC_T7X_Position1	aGGTCTCagcatTTTgggGGCatggggtctgcacttcgtct
Rv_HheC_T7R	tGGTCTCAaatgcttaacgttGCGtacaattgcggttgacatatggct
Rv_HheC_T7H	tGGTCTCAaatgcttaacgttATGtacaattgcggttgacatatggct
Rv_HheC_T7K	tGGTCTCAaatgcttaacgttTTTtacaattgcggttgacatatggct
Rv_HheC_T7D	tGGTCTCAaatgcttaacgttATCtacaattgcggttgacatatggct
Rv_HheC_T7E	tGGTCTCAaatgcttaacgttTTCtacaattgcggttgacatatggct
Rv_HheC_T7S	tGGTCTCAaatgcttaacgttCGAtacaattgcggttgacatatggct
Rv_HheC_T7N	tGGTCTCAaatgcttaacgttGTTtacaattgcggttgacatatggct
Rv_HheC_T7Q	tGGTCTCAaatgcttaacgttCTGtacaattgcggttgacatatggct
Rv_HheC_T7C	tGGTCTCAaatgcttaacgttGCAtacaattgcggttgacatatggct
Rv_HheC_T7G	tGGTCTCAaatgcttaacgttGCCtacaattgcggttgacatatggct
Rv_HheC_T7P	tGGTCTCAaatgcttaacgttCGGtacaattgcggttgacatatggct
Rv_HheC_T7A	tGGTCTCAaatgcttaacgttCGCtacaattgcggttgacatatggct
Rv_HheC_T7V	tGGTCTCAaatgcttaacgttCACtacaattgcggttgacatatggct
Rv_HheC_T7I	tGGTCTCAaatgcttaacgttAATtacaattgcggttgacatatggct
Rv_HheC_T7L	tGGTCTCAaatgcttaacgttCAGtacaattgcggttgacatatggct
Rv_HheC_T7M	tGGTCTCAaatgcttaacgttCATtacaattgcggttgacatatggct
Rv_HheC_T7Y	tGGTCTCAaatgcttaacgttATAtacaattgcggttgacatatggct
Rv_HheC_T7W	tGGTCTCAaatgcttaacgttCCAtacaattgcggttgacatatggct
Rv_HheC_T7F	tGGTCTCAaatgcttaacgttAAAtacaattgcggttgacatatggct
Rv_HheC_F12C	CCCCATTCCCCCGCAATGCTTAACG
Rv_HheC_F12D	CCCCATTCCCCCATCATGCTTAACGTTTG
Rv_HheC_F12G	GACCCCATTCCCCCGCCATGCTTAACGTTTG
Rv_HheC_F12I	CCCATTCCCCCAATATGCTTAACGTTTG
Rv_HheC_F12L	GACCCCATTCCCCCCAGATGCTTAACGTTTG
Rv_HheC_F12N	CCCCATTCCCCCATTATGCTTAACGTTTG
Rv_HheC_F12P	GACCCCATTCCCCCCGGATGCTTAACGTTTG
Rv_HheC_F12R	CCCCATTCCCCCACGATGCTTAACGTTTG
Rv_HheC_F12S	GACCCCATTCCCCCGCTATGCTTAACGTTTG
Rv_HheC_F12T	GACCCCATTCCCCCGGTATGCTTAACGTTTG
Rv_HheC_F12X/G14X	tGGTCTCAaatgcttaacgttTGTtacaattgcggttgacatatggct
Fw_HheC_F12R	aGGTCTCagcatCGCgggGGCatggggtctgcacttcgtct
Fw_HheC_F12H	aGGTCTCagcatCATgggGGCatggggtctgcacttcgtct
Fw_HheC_F12K	aGGTCTCagcatAAAgggGGCatggggtctgcacttcgtct
Fw_HheC_F12D	aGGTCTCagcatGATgggGGCatggggtctgcacttcgtct
Fw_HheC_F12E	aGGTCTCagcatGAAgggGGCatggggtctgcacttcgtct
Fw_HheC_F12S	aGGTCTCagcatAGCgggGGCatggggtctgcacttcgtct
Fw_HheC_F12T	aGGTCTCagcatACCgggGGCatggggtctgcacttcgtct
Fw_HheC_F12N	aGGTCTCagcatAACgggGGCatggggtctgcacttcgtct

Fw_HheC_F12Q	aGGTCTCagcatCAGgggGGCatggggtctgcacttcgtct
Fw_HheC_F12C	aGGTCTCagcatTGCgggGGCatggggtctgcacttcgtct
Fw_HheC_F12G	aGGTCTCagcatGGCgggGGCatggggtctgcacttcgtct
Fw_HheC_F12P	aGGTCTCagcatCCGgggGGCatggggtctgcacttcgtct
Fw_HheC_F12A	aGGTCTCagcatGCGgggGGCatggggtctgcacttcgtct
Fw_HheC_F12V	aGGTCTCagcatGTGgggGGCatggggtctgcacttcgtct
Fw_HheC_F12I	aGGTCTCagcatATTgggGGCatggggtctgcacttcgtct
Fw_HheC_F12L	aGGTCTCagcatTTAgggGGCatggggtctgcacttcgtct
Fw_HheC_F12M	aGGTCTCagcatATGgggGGCatggggtctgcacttcgtct
Fw_HheC_F12W	aGGTCTCagcatTGGgggGGCatggggtctgcacttcgtct
Fw_HheC_F12Y	aGGTCTCagcatTATgggGGCatggggtctgcacttcgtct
Fw_HheC_G14R	aGGTCTCagcatTTTgggCGCatggggtctgcacttcgtct
Fw_HheC_G14H	aGGTCTCagcatTTTgggCATatggggtctgcacttcgtct
Fw_HheC_G14K	aGGTCTCagcatTTTgggAAAatggggtctgcacttcgtct
Fw_HheC_G14D	aGGTCTCagcatTTTgggGATatggggtctgcacttcgtct
Fw_HheC_G14E	aGGTCTCagcatTTTgggGAAatggggtctgcacttcgtct
Fw_HheC_G14S	aGGTCTCagcatTTTgggTCGatggggtctgcacttcgtct
Fw_HheC_G14T	aGGTCTCagcatTTTgggACCatggggtctgcacttcgtct
Fw_HheC_G14N	aGGTCTCagcatTTTgggAACatggggtctgcacttcgtct
Fw_HheC_G14Q	aGGTCTCagcatTTTgggCAGatggggtctgcacttcgtct
Fw_HheC_G14C	aGGTCTCagcatTTTgggTGCatggggtctgcacttcgtct
Fw_HheC_G14P	aGGTCTCagcatTTTgggCCGatggggtctgcacttcgtct
Fw_HheC_G14A	aGGTCTCagcatTTTgggGCGatggggtctgcacttcgtct
Fw_HheC_G14V	aGGTCTCagcatTTTgggGTGatggggtctgcacttcgtct
Fw_HheC_G14I	aGGTCTCagcatTTTgggATTatggggtctgcacttcgtct
Fw_HheC_G14L	aGGTCTCagcatTTTgggTTAatggggtctgcacttcgtct
Fw_HheC_G14M	aGGTCTCagcatTTTgggATGatggggtctgcacttcgtct
Fw_HheC_G14Y	aGGTCTCagcatTTTgggTATatggggtctgcacttcgtct
Fw_HheC_G14F	aGGTCTCagcatTTTgggTTTatggggtctgcacttcgtct
Fw_HheC_G14W	aGGTCTCagcatTTTgggTGGatggggtctgcacttcgtct
Fw_HheG_T13X	aGGTCTCccggtTATgttGGCccggcactggcacgtaccatg
Rv_HheG_T13R	tGGTCTCAaccggttgccatCCTaatcagtgcaaccggacgattttctg
Rv_HheG_T13H	tGGTCTCAaccggttgccatATGaatcagtgcaaccggacgattttctg
Rv_HheG_T13K	tGGTCTCAaccggttgccatTTTaatcagtgcaaccggacgattttctg
Rv_HheG_T13D	tGGTCTCAaccggttgccatATCaatcagtgcaaccggacgattttctg
Rv_HheG_T13E	tGGTCTCAaccggttgccatTTCaatcagtgcaaccggacgattttctg
Rv_HheG_T13S	tGGTCTCAaccggttgccatCGAaatcagtgcaaccggacgattttctg
Rv_HheG_T13N	tGGTCTCAaccggttgccatGTTaatcagtgcaaccggacgattttctg
Rv_HheG_T13Q	tGGTCTCAaccggttgccatCTGaatcagtgcaaccggacgattttctg
Rv_HheG_T13C	tGGTCTCAaccggttgccatGCAaatcagtgcaaccggacgattttctg
Rv_HheG_T13G	tGGTCTCAaccggttgccatGCCaatcagtgcaaccggacgattttctg
Rv_HheG_T13P	tGGTCTCAaccggttgccatCGGaatcagtgcaaccggacgattttctg
Rv_HheG_T13A	tGGTCTCAaccggttgccatCGCaatcagtgcaaccggacgattttctg
Rv_HheG_T13V	tGGTCTCAaccggttgccatCACaatcagtgcaaccggacgattttctg
Rv_HheG_T13I	tGGTCTCAaccggttgccatAATaatcagtgcaaccggacgattttctg
Rv_HheG_T13L	tGGTCTCAaccggttgccatTAAaatcagtgcaaccggacgattttctg
Rv_HheG_T13M	tGGTCTCAaccggttgccatCATaatcagtgcaaccggacgattttctg
Rv_HheG_T13Y	tGGTCTCAaccggttgccatATAaatcagtgcaaccggacgattttctg
Rv_HheG_T13W	tGGTCTCAaccggttgccatCCAaatcagtgcaaccggacgattttctg
Rv_HheG_T13F	tGGTCTCAaccggttgccatAAAaatcagtgcaaccggacgattttctg
Rv_HheG_Y18X/G20X	tGGTCTCAaccggttgccatCGTaatcagtgcaaccggacgattttctg
Fw_HheG_Y18R	aGGTCTCccggtAGGgttGGCccggcactggcacgtaccatg
Fw_HheG_Y18H	aGGTCTCccggtCATgttGGCccggcactggcacgtaccatg
Fw_HheG_Y18K	aGGTCTCccggtAAAgttGGCccggcactggcacgtaccatg
Fw_HheG_Y18D	aGGTCTCccggtGATgttGGCccggcactggcacgtaccatg
Fw_HheG_Y18E	aGGTCTCccggtGAAgttGGCccggcactggcacgtaccatg
Fw_HheG_Y18S	aGGTCTCccggtAGCgttGGCccggcactggcacgtaccatg

Fw_HheG_Y18T	aGGTCTCccggtACCgttGGCccggcactggcacgtaccatg
Fw_HheG_Y18N	aGGTCTCccggtAACgttGGCccggcactggcacgtaccatg
Fw_HheG_Y18Q	aGGTCTCccggtCAGgttGGCccggcactggcacgtaccatg
Fw_HheG_Y18C	aGGTCTCccggtTGCgttGGCccggcactggcacgtaccatg
Fw_HheG_Y18G	aGGTCTCccggtGGCgttGGCccggcactggcacgtaccatg
Fw_HheG_Y18P	aGGTCTCccggtCCGgttGGCccggcactggcacgtaccatg
Fw_HheG_Y18A	aGGTCTCccggtGCGgttGGCccggcactggcacgtaccatg
Fw_HheG_Y18V	aGGTCTCccggtGTGgttGGCccggcactggcacgtaccatg
Fw_HheG_Y18I	aGGTCTCccggtATTgttGGCccggcactggcacgtaccatg
Fw_HheG_Y18L	aGGTCTCccggtTTAgttGGCccggcactggcacgtaccatg
Fw_HheG_Y18M	aGGTCTCccggtATGgttGGCccggcactggcacgtaccatg
Fw_HheG_Y18W	aGGTCTCccggtTGGgttGGCccggcactggcacgtaccatg
Fw_HheG_Y18F	aGGTCTCccggtTTTgttGGCccggcactggcacgtaccatg
Fw_HheG_G20R	aGGTCTCccggtTATgttCGCccggcactggcacgtaccatg
Fw_HheG_G20H	aGGTCTCccggtTATgttCATccggcactggcacgtaccatg
Fw_HheG_G20K	aGGTCTCccggtTATgttAAAccggcactggcacgtaccatg
Fw_HheG_G20D	aGGTCTCccggtTATgttGATccggcactggcacgtaccatg
Fw_HheG_G20E	aGGTCTCccggtTATgttGAAccggcactggcacgtaccatg
Fw_HheG_G20S	aGGTCTCccggtTATgttAGCccggcactggcacgtaccatg
Fw_HheG_G20T	aGGTCTCccggtTATgttACCccggcactggcacgtaccatg
Fw_HheG_G20N	aGGTCTCccggtTATgttAACccggcactggcacgtaccatg
Fw_HheG_G20Q	aGGTCTCccggtTATgttCAGccggcactggcacgtaccatg
Fw_HheG_G20C	aGGTCTCccggtTATgttTGCccggcactggcacgtaccatg
Fw_HheG_G20P	aGGTCTCccggtTATgttCCGccggcactggcacgtaccatg
Fw_HheG_G20A	aGGTCTCccggtTATgttGCGccggcactggcacgtaccatg
Fw_HheG_G20V	aGGTCTCccggtTATgttGTGccggcactggcacgtaccatg
Fw_HheG_G20I	aGGTCTCccggtTATgttATTccggcactggcacgtaccatg
Fw_HheG_G20L	aGGTCTCccggtTATgttTTAccggcactggcacgtaccatg
Fw_HheG_G20M	aGGTCTCccggtTATgttATGccggcactggcacgtaccatg
Fw_HheG_G20Y	aGGTCTCccggtTATgttTATccggcactggcacgtaccatg
Fw_HheG_G20F	aGGTCTCccggtTATgttTTTccggcactggcacgtaccatg
Fw_HheG_Y18W	aGGTCTCccggtTATgttTGGccggcactggcacgtaccatg

Table S7. Golden gate mutagenesis reaction compounds and their final amounts/concentrations in a total volume of 50 µL.

Compound	concentrations$_{final}$
Forward/reverse mutagenic primer	Each 0.50 µM
pET28a(+)-*hheC* or pET28a(+)-*hheG*	1.00 ng
Q5 HotStart High-Fidelity DNA-Polymerase	0.01 U
dNTPs	200 µM
Q5 reaction buffer	1x

Optimization of the BTB-based pH assay for quantification of epoxide ring opening activity

Theory for assay development and derivation of equations

To assay HHDHs for the formation or ring opening of epoxides, we sought to develop an indicator-based assay. Ideally such an approach should enable high-throughput experimentation in multiwell plates, be compatible the forward and reverse reaction as well as various HHDHs and nucleophiles, be sensitive enough for kinetic experiments and be relatively insensitive to experimental errors such as routine pipetting inaccuracies or spectrometer noise. Building on the qualitative indicator-based assay reported by Gul and colleagues, we developed a physicochemical framework which derives highly quantitative reaction rates from pairs of UV signals.[2] This approach was driven by the retrosynthetic realization that both types of reactions catalyzed by HHDHs feature the conversion of a strong acid to a weak acid, or vice versa (e.g. cyclohexene oxide and azide, a strong acid, are converted to 2-azidocyclohexanol, an alcohol, which is a weak acid). This interchange effects a change in the free proton concentration and, in turn, in the pH value of the reaction mixture (which would depend on the buffer pK_a, the buffer strength and the starting pH). The use of an indicator reflecting this change in pH should then allow a quantitative monitoring of this change of pH. Thus, in a forward sense, we employed dual-wavelength UV-spectroscopic monitoring of an indicator to assess the pH of the reaction mixture at various time points and translated this change in pH to a concentration of protons produced/consumed via acid-base equilibria of the buffer system.

For this approach, we enlisted bromothymol blue (3,3-Bis[3-bromo-4-hydroxy-2-methyl-5-(propan-2-yl)phenyl]-2,1λ6-benzoxathiole-1,1(3H)-dione, CAS 76-59-5) as an indicator. Bromothymol blue i) has a pK_a of around 7.1 (we measured it to 7.15 ± 0.02 by analysis of its UV absorption spectra over different pH values in 2 mM citrate/MOPS/glycine buffer), ii) has a broad UV absorption profile with a λ_{max} of its anion at 616 nm and four isosbestic points of deprotonation (254, 282, 324, and 499 nm) (Figure S16), of which the highest one is well suited for normalization purposes since it is far outside the absorption range of typical artefacts such as absorption by proteins or salts and iii) has very high extinction coefficients (ca. 7 mM^{-1} cm^{-1} at 499 nm and 50 mM^{-1} cm^{-1} at 616 nm for the anion), enabling its use under very dilute conditions, although it should be noted that our approach based on isosbestic point normalization does not take concentration into account.

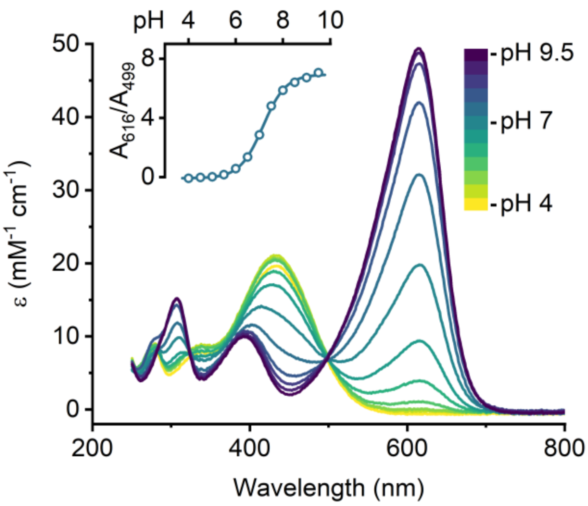

Figure S16. UV absorption spectra of BTB at different pH values, showing the four isosbestic points and λ_{max} of its anion at 616 nm.

The mathematical considerations underlying our assay approach do make some simplifying assumptions. First, we assumed that only one buffer component is present in the mixture. This implies that all other components (including the indicator, the protein(s) and other impurities) do not act as buffers. From a mathematical perspective, this means that the concentration of the indicator is equal to zero, which is, obviously, a slight oversimplification, but it does hold up for indicator concentrations far below the buffer concentration. We further assumed that stoichiometry holds true and that the mass balance of the buffer is perfectly distributed between an acidic and a basic species. Lastly, we assumed that all changes in the apparent pH were exclusively caused by the enzymatic reaction in question and that off-model effects such as precipitation of poorly soluble neutral species, pH shifts

caused by (de-)protonation of protein residues, monomer leeching of the multiwell plates or latent pH shifts do not occur. Thus, the following basal relationships apply.

$$BH^+ \leftrightharpoons B + H^+ \tag{S1}$$
$$IH^+ \leftrightharpoons I + H^+ \tag{S2}$$
$$[I] + [IH^+] \ll [B] + [BH^+] \tag{S3}$$
$$[B] = [B]_0 - \Delta[H^+] \tag{S4}$$
$$[BH^+] = [BH^+]_0 + \Delta[H^+] \tag{S5}$$
$$\Delta[H^+]_0 = 0 \tag{S6}$$

where B is the neutral (or deprotonated) species of the buffer with the equilibrium concentration $[B]$ and the initial concentration $[B]_0$, BH^+ is the protonated species of the buffer with the equilibrium concentration $[BH^+]$ and the initial concentration $[BH^+]_0$, H^+ is the proton with the concentration $[H^+]$ (and the concentration change $\Delta[H^+]$), I the neutral (or deprotonated) species of the indicator with the equilibrium concentration $[I]$, and IH^+ is the protonated species of the indicator with the equilibrium concentration $[IH^+]$. Herein, all concentrations can be treated in arbitrary molar concentrations. For the sake of practicality in biochemical settings, we used mM throughout our work.

Thus, our approach considers two acid-base equilibria (that of the buffer and that of the indicator), which can be described by distinct terms. The two are intrinsically linked by the pH value of the solution, while the equilibrium of the buffer dictates to what extent the production/consumption of protons influences the net pH, while the equilibrium of the indicator grants UV-spectroscopic access to said net pH of the solution.

The pH of the solution is thus available through the distribution of the neutral and anionic species of the indicator. Following the fundamental relationship

$$\alpha_I = \frac{10^{pH-pK_{a,I}}}{1 + 10^{pH-pK_{a,I}}} \tag{S7}$$

and rearrangement to

$$pH = log\left(\frac{\alpha_I\ 10^{pK_{a,I}}}{1-\alpha_I}\right) \tag{S8}$$

directly yields the pH as a function of the degree of deprotonation of the indicator, α_I, and the pK_a value of the indicator, $pK_{a,I}$ (all of these values are dimensionless). Herein, α is given by

$$\alpha_I = \frac{[I]}{[I] + [IH^+]} \tag{S9}$$

with definitions from above. Through analysis of the UV absorption spectral of bromothymol blue, α_I is directly accessible through two absorption values as

$$\alpha_I = 0.1424\ \frac{A_{616}}{A_{499}} \tag{S10}$$

where A_{616} is the blank-corrected absorption at the reference wavelength λ_{max} (616 nm) and A_{499} is the blank-corrected absorption at the isosbestic point of deprotonation (499 nm). The preceding empirical factor of 0.1424 normalizes the reference values for $\alpha_I \approx 1$ (A_{616}/A_{499} = 6.94 ± 0.08) and $\alpha_I \approx 0$ (A_{616}/A_{499} = −0.08 ± 0.02) to a scale between 1 and 0 (1/7.02 = 0.1424). All of these values are dimensionless and are given in arbitrary units.

Analysis of the acid-base equilibria of the buffer then enables a translation of shifts in the apparent pH accessible by UV spectroscopy into a change in proton concentration. The distribution of the different buffer species defined above follows

$$pH = pK_{a,B} + log\left(\frac{[B]}{[BH^+]}\right) \tag{S11}$$

which can be supplemented with the terms from equations (S4) and (S5) to yield

$$pH = pK_{a,B} + log\left(\frac{[B]_0 - \Delta[H^+]}{[BH^+]_0 + \Delta[H^+]}\right) \tag{S12}$$

which already contains a change in proton concentration, as well as the initial concentrations of the individual buffer species. Here, $pK_{a,B}$ is the dimensionless pK_a value of the buffer. Rearrangement of this term then yields

$$10^{pH} = 10^{pK_{a,B}} + \frac{[B]_0 - \Delta[H^+]}{[BH^+]_0 + \Delta[H^+]} \tag{S13}$$

which can be further rearranged to

$$10^{pH}[BH^+]_0 + 10^{pH}\Delta[H^+] = 10^{pK_{a,B}}[B]_0 - 10^{pK_{a,B}}\Delta[H^+] \tag{S14}$$

and simplified to give the change in proton concentration as a function of the pH and the initial distribution of the different species as

$$\Delta[H^+] = \frac{10^{pK_{a,B}}[B]_0 - 10^{pH}[BH^+]_0}{10^{pH} + 10^{pK_{a,B}}} \tag{S15}$$

The necessary initial concentrations $[B]_0$ and $[BH^+]_0$ are available through a similar analysis as described in equations (S7) and (S9), by relying on the mass balances of the system. Thus, the initial distribution of the different buffer species follows

$$\alpha_{0,B} = \frac{[BH^+]_0}{[BH^+]_0 + [B]_0} = \frac{10^{pH_0 - pK_{a,B}}}{1 + 10^{pH_0 - pK_{a,B}}} = \frac{[BH^+]_0}{x_0} \tag{S16}$$

where $\alpha_{0,B}$ is the dimensionless degree of protonation of the buffer, $pK_{a,B}$ is the pK_a value of the buffer, x_0 is the total buffer concentration (in arbitrary molar concentrations), and pH_0 is the initial pH. As such, the initial distribution of the charged buffer species is given by

$$[BH^+]_0 = x_0 \frac{10^{pH_0 - pK_{a,B}}}{1 + 10^{pH_0 - pK_{a,B}}} \tag{S17}$$

$$[B]_0 = x_0 - [BH^+]_0 \tag{S18}$$

Mathematical workflow for the indicator assay

From an end user's perspective, the above equations can be summarized as follows.

First, the initial concentrations of the buffer species can be calculated as

$$[BH^+]_0 = x_0 \frac{10^{pH_0 - pK_{a,B}}}{1 + 10^{pH_0 - pK_{a,B}}} \tag{S17}$$

$$[B]_0 = x_0 - [BH^+]_0 \tag{S18}$$

with

pH_0	starting pH of the reaction mixture, typically equivalent to the pH value of the buffer
$pK_{a,B}$	pK_a value of the buffer used (e.g. 7.2 for MOPS or phosphate, 7.5 for HEPES, all at 25 °C)
x_0	total buffer concentration (e.g. in mM)

Secondly, a measurement of two UV signals for the indicator gives a (current) pH value for the reaction mixture. At the initiation of the reaction, this value should be equal or close to the assumed starting pH, at later timepoints, this value may deviate significantly.

$$pH = log\left(\frac{\alpha_I\ 10^{pK_{a,I}}}{1-\alpha_I}\right) \tag{S8}$$

with

$$\alpha_I = 0.1424\ \frac{A_{616}}{A_{499}} \tag{S10}$$

with

pH_0	starting pH of the reaction mixture, typically equivalent to the pH value of the buffer
$pK_{a,I}$	pK_a value of the indicator used (e.g. 7.15 for bromothymol blue at 25 °C)
A_{616}	blank-corrected absorption at 616 nm (AU)
A_{499}	blank-corrected absorption at 499 nm (AU)

Thirdly, substitution of $[B]_0$ (from equation (S18)), $[BH^+]_0$ (from equation (S17)), and pH (from equation (S8)) gives the change in proton concentration via

$$\Delta[H^+] = \frac{10^{pK_{a,B}}[B]_0 - 10^{pH}[BH^+]_0}{10^{pH} + 10^{pK_{a,B}}} \tag{S15}$$

The unit of $\Delta[H^+]$ will herein be equivalent to the one used for x_0. It should also be noted that depending on which way α_I and α_B were defined, equation (S15) may return negative or positive values. Either way, the absolute of this change will reflect reaction progress.

Example values

To give some exemplary values, we briefly consider a representative case in a MOPS-buffered system. In a 20 mM MOPS buffer at pH 7 ($pK_{a,B}$ = 7.2), x_0 = 20 mM with $[B]_0$ = 7.74 mM and $[BH^+]_0$ = 12.26 mM. Using bromothymol blue as an indicator, this should give an A_{616}/A_{499}-ratio of around 2.91 (using blank-corrected values, for instance A_{616} = 0.723 with a background of 0.036 and A_{499} = 0.276 with a background of 0.040). In that case, α_I = 0.414. Starting from this initial pH, a proton-consuming reaction may give the following A_{616} and A_{499} values, which can be translated into $\Delta[H^+]$ with the equations above.

Table S8. Exemplary absorption data and their translation into a change in proton concentration.

Timepoint	A_{499} (AU)	A_{499} blank (AU)	A_{616} (AU)	A_{616} blank (AU)	α_I	pH	$\lvert\Delta[H^+]\rvert$ (mM)
0	0.276	0.040	0.723	0.036	0.414	7.000	0.00
1	0.269	0.040	0.739	0.037	0.436	7.039	0.43
2	0.267	0.039	0.758	0.036	0.451	7.064	0.72
3	0.267	0.038	0.776	0.035	0.461	7.082	0.91
4	0.266	0.037	0.789	0.036	0.468	7.095	1.06

Gas chromatography (GC)

To determine conversion or enantiomeric excess, GC analysis (GC2010 plus, Shimadzu, Duisburg, Germany) was performed after extraction of reaction samples with an equal volume of *tert*-butyl methyl ether (TBME) and drying of the organic phase over anhydrous $MgSO_4$. Achiral separation was carried out on an OPTIMA 5 MS column (30 m length, 0.25 mm inner diameter, 0.25 µm film thickness, Macherey Nagel, Düren, Germany), whereas chiral analysis was performed using a HYDRODEX γ-DIMOM column (25 m length, 0.25 mm inner diameter, 0.25 µm film thickness, Macherey Nagel) with 40 cm/s hydrogen carrier gas flow. Injector and detector temperature were set at 300 °C for achiral GC analysis. For chiral GC analysis, instead, injector and detector temperature were set at 200 °C and 250 °C, respectively. Table S8 lists all applied temperature programs and respective retentions times of substrates and products used in this study.

Table S9. GC temperature programs and retention times of substrates and products used in this study.

Compound	Temperature program	Retention time
**Achiral separation (OPTIMA 5 MS)**		
cyclohexene oxide (**2**)	7.5 min at 110 °C // heating with 50 °C/min to 295 °C	2.01 min
2-azido-1-cyclohexanol (**2a**)		5.42 min
(+)-*cis*-limonene oxide (**5**)	1 min at 80 °C // heating with 10 °C/min to 160 °C // heating with 20 °C/min to 300 °C	6.90 min
(+)-*trans*-limonene oxide (**5**)		7.01 min
2-azido-1-methyl-4-(prop-1-en-2-yl)cyclohexan-1-ol (**5a.1**)		10.5 min
2-azido-2-methyl-5-(prop-1-en-2-yl)cyclohexan-1-ol (**5a.2**)		11.0 min
styrene oxide (**3**)	1 min at 80 °C // heating with 10 °C/min to 160 °C // heating with 20 °C/min to 300 °C	5.90 min
2-azido-2-phenylethanol (**3a**)		10.0 min
trans-1-phenylpropylene oxide (**6**)		6.60 min
1-azido-1-phenylpropan-2-ol (**6a**)		10.1 min
glycidyl phenyl ether (**4**)	1 min at 80 °C // heating with 10 °C/min to 160 °C // heating with 20 °C/min to 300 °C	9.10 min
3-phenoxypropane-1,2-diol (**4h**)		6.80 min
1-azido-3-phenoxypropan-2-ol (**4a**)		12.1 min
1-nitro-3-phenoxypropan-2-ol (**4b**)		11.5 min
1-chloro-3-phenoxypropan-2-ol (**4f**)		11.0 min
1-bromo-3-phenoxypropan-2-ol (**4g**)		11.9 min
1-(iso)thiocyanato-3-phenoxypropan-2-ol (**4e.1**/**4e.2**)		10.8 min
5-(phenoxymethyl)oxazolidin-2-one (**4d**)		11.9 min
3-hydroxy-4-phenoxybutanenitrile (**4c**)		12.1 min
1-(iso)thiocyanato-3-phenoxypropan-2-ol (**4e.1**/**4e.2**)		14.4 min
epichlorohydrin (**1**)	1 min at 40 °C // heating with 10 °C/min to 120 °C // heating with 20 °C/min to 300 °C	3.80 min
1,3-dichloro-2-propanol (**1f**)		6.60 min
1-azido-3-chloro-2-propanol (**1a**)		10.5 min
**Chiral separation (HYDRODEX γ-DIMOM)**		
cyclohexene oxide (**2**)	40 min at 100 °C // heating with 10 °C/min to 200 °C	3.10 min
(1*S*, 2*S*)-2-azidocyclohexan-1-ol (**2a**)		34.1 min
(1*R*, 2*R*)-2-azidocyclohexan-1-ol (**2a**)		35.4 min
(*R/S*)-epichlorohydrin (**1**)	15 min at 90 °C, // heating with 10 °C/min to 140 °C // 10 min at 140 °C // heating with 0.5 °C/min to 150 °C // 10 min at 150 °C // heating with 10 °C/min to 200 °C	2.01 min
(*R*)-1-azido-3-chloro-2-propanol (**1a**)		20.0 min
(*S*)-1-azido-3-chloro-2-propanol (**1a**)		20.2 min
(*R*)-styrene oxide (**3**)		11.2 min
(*S*)-styrene oxide (**3**)		11.7 min
(*R*)-2-azido-2-phenylethan-1-ol (**3a**)		33.3 min
(*S*)-2-azido-2-phenylethan-1-ol (**3a**)		33.7 min
(*R*)-2-azido-1-phenylethan-1-ol (**3a**)		43.0 min
(*S*)-2-azido-1-phenylethan-1-ol (**3a**)		44.0 min

Product characterization

For structure validation of newly produced β-substituted alcohols starting from epoxide **4**, preparative-scale reactions were performed.[3] Reactions in a total volume of 30 mL containing 50 mM Tris·SO_4 buffer, pH 7.0, 20 mM glycidyl phenyl ether (**4**), 20 mM nucleophile **a**–**e** and 400 µg HheC were carried out at 30 °C and 700 rpm for 24 h. Afterwards, the reaction mixtures were extracted twice using *tert*-butyl methyl ether and the combined organic extracts were dried over $MgSO_4$. Successful product formation was analyzed *via* achiral GC. The solvent was evaporated *in vacuo* and the products were purified by flash chromatography on silica gel (silica gel 60, particle size 0.040-0.063 mm, mesh 230–440 ASTM, Fluka) using different solvent systems. Technical-grade solvents for chromatography were distilled before use.

Thin layer chromatography (TLC) was performed using silica-coated plates Polygram SIL G/UV254 (Macherey & Nagel). The starting material, glycidyl phenyl ether (**4**), has R_f = 0.52 (1:4 EtOAc/Cyclohexane), R_f = 0.66 (1:3 EtOAc/Cyclohexane), and R_f = 0.9 (EtOAc). Both the starting material and all products stain well in CAM stain (1 g $Ce(IV)(SO_4)_2$ and 5 g $(NH_4)_6Mo_4O_7$ in 90 mL water and 10 mL concentrated H_2SO_4) and are weakly visible by UV (254 nm). GC-HRMS analysis was performed using an Agilent 6890 gas chromatograph equipped with a 30 m analytical ZB1-MS column (Phenomenex, 30 m x 0.25 mm ID, tf=0.25 µm). For sample injection, a split injection port at 270 °C and a split ratio of 10:1 was used. The temperature program was 50 °C (3 min)-10 °C min^{-1}-310 °C (3 min) and helium carrier gas was set to 1.0 ml min^{-1} flow rate. A JMS-T100GC (GCAccuTOF, JEOL, Japan) time of flight mass spectrometer in electron ionization (EI) mode at 70 eV and JEOL MassCenter™ workstation software was used. The source and transfer line temperatures were set at 200 °C and 270 °C, respectively. The detector voltage was set at 2000 V. The acquisition range was from m/z 41 to 600 with a spectrum recording interval of 0.4 s. The system was tuned with perfluoro kerosene (PFK) to achieve a resolution of 5,000 (FWHM) at m/z 292.9824.

NMR spectra were recorded on a Bruker AV300, Bruker AVIII400, Bruker AVIIIHD500 (with CryoProbe) and Bruker AVANCE III 600 instrument using TMS as the internal reference and analysis was performed in MestreNova. Data are reported as follows: chemical shift (ppm), integration, multiplicity (s = singlet, d = doublet, t = triplet, q = quartet, m = multiplet), coupling constant(s) (Hz), and assignment.

1-azido-3-phenoxypropan-2-ol (**4a**),
Following the general procedure with 90.1 mg **4** and azide (**a**) gave 98.9 mg crude product (yellow oil). Purification by flash chromatography with 1:4 EtOAc/Cyclohexane yielded 70 mg **4a**.

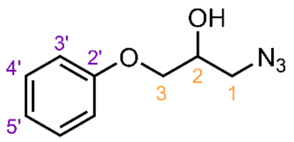

Physical state palely yellow oil

^{1}H NMR (500 MHz, $CDCl_3$) δ 7.31−7.26 (2H, m, H-4′), 6.98 (1H, tt, *J* = 1.1, 7.3 Hz, H-5′), 6.92−6.88 (2H, m, H-3′), 4.17−4.12 (1H, m, H-2), 3.99 (1H, s, H-3), 3.98 (1H, d, *J* = 1.1 Hz, H-3), 3.52 (1H, dd, *J* = 4.6, 12.7 Hz, H-1), 3.47 (1H, dd, *J* = 6.1, 12.7 Hz, H-1)

^{13}C NMR (125 MHz, $CDCl_3$) δ 158.2 (Cq, C-2′), 129.6 (CH, C-4′), 121.5 (CH, C-5′), 114.6 (CH, C-3′), 69.3 (CH, C-2), 69.0 (CH_2, C-3), 53.4 (CH_2, C-1)

HRMS m/z calc. 193.085 for $C_9H_{11}N_3O_2$, found 193.080

TLC R_f = 0.36 (1:4 EtOAc/Cyclohexane)

1-nitro-3-phenoxypropan-2-ol (**4b**)
Following the general procedure with 90.1 mg **4** and nitrite (**b**) gave 56.2 mg crude product (orange oil). Purification by flash chromatography with 1:3 EtOAc/Cyclohexane yielded 13.1 mg **4b**.

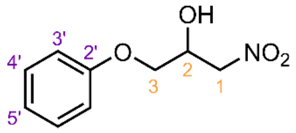

Physical state yellowish oil

^{1}H NMR (500 MHz, $CDCl_3$) δ 7.32−7.27 (2H, m, H-4′), 6.99 (1H, tt, *J* = 1.1, 7.4 Hz, H-5′), 6.92−6.88 (2H, m, H-3′), 4.34−4.27 (1H, m, H-3), 4.02 (1H, d, *J* = 5.2 Hz, H-4), 2.75 (1H, dd, *J* = 5.6, 17.0 Hz, H-2), 2.69 (1H, dd, *J* = 6.6, 16.9 Hz, H-2)

^{13}C NMR (125 MHz, $CDCl_3$) δ 157.9 (Cq, C-2′), 129.6 (CH, C-4′), 121.7 (CH, C-5′), 117.1 (Cq, C-1), 114.5 (CH, C-3′), 69.8 (CH, C-4), 66.2 (CH_2, C-3), 22.6 (CH_2, C-2)

HRMS m/z calc. 197.070 for $C_9H_{11}NO_4$, found 197.070

TLC R_f = 0.24 (1:4 EtOAc/Cyclohexane)
R_f = 0.55 (1:3 EtOAc/Cyclohexane)

3-phenoxypropane-1,2-diol (**4h**)
Following the general procedure with 90.1 mg **4** and nitrite (**b**) gave 56.2 mg crude product (orange oil). Purification by flash chromatography with 1:3 EtOAc/Cyclohexane→EtOAc yielded 25.4 mg **4h**.

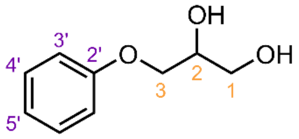

Physical state fluffy orange solid (brownish oil at 40 °C)

^{1}H NMR (500 MHz, $CDCl_3$) δ 7.31−7.24 (2H, m, H-4′), 6.97 (1H, tt, *J* = 1.1, 7.3 Hz, H-5′), 6.92−6.88 (2H, m, H-3′), 4.11−4.08 (1H, m, H-2), 4.03 (1H, s, H-3), 4.02 (1H, d, *J* = 2.2 Hz, H-3), 3.83 (1H, dd, *J* = 3.7, 11.5 Hz, H-1), 3.73 (1H, dd, *J* = 5.7, 11.5 Hz, H-1)

^{13}C NMR (125 MHz, $CDCl_3$) δ 158.4 (Cq, C-2′), 129.6 (CH, C-4′), 121.3 (CH, C-5′), 114.6 (CH, C-3′), 70.5 (CH, C-2), 69.0 (CH_2, C-3), 63.7 (CH_2, C-1)

HRMS m/z calc. 168.080 for $C_9H_{12}O_3$, found 168.080

TLC R_f = 0.02 (1:4 EtOAc/Cyclohexane)
R_f = 0.09 (1:3 EtOAc/Cyclohexane)

3-hydroxy-4-phenoxybutanenitrile (**4c**)
Following the general procedure with 90.1 mg **4** and cyanide (**c**) gave 68.7 mg crude product (yellow oil). Purification by flash chromatography with 1:3 EtOAc/Cyclohexane yielded 47.6 mg **4c**.

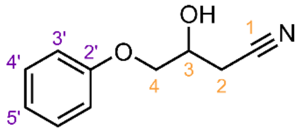

Physical state highly viscous colorless oil

^{1}H NMR (500 MHz, $CDCl_3$) δ 7.32−7.27 (2H, m, H-4′), 6.99 (1H, tt, *J* = 1.1, 7.4 Hz, H-5′), 6.92−6.88 (2H, m, H-3′), 4.34−4.27 (1H, m, H-3), 4.02 (1H, d, *J* = 5.2 Hz, H-4), 2.75 (1H, dd, *J* = 5.6, 17.0 Hz, H-2), 2.69 (1H, dd, *J* = 6.6, 16.9 Hz, H-2)

^{13}C NMR (125 MHz, $CDCl_3$) δ 157.9 (Cq, C-2′), 129.6 (CH, C-4′), 121.7 (CH, C-5′), 117.1 (Cq, C-1), 114.5 (CH, C-3′), 69.8 (CH, C-4), 66.2 (CH_2, C-3), 22.6 (CH_2, C-2)

HRMS m/z calc. 177.080 for $C_{10}H_{11}NO_2$, found 177.080

TLC R_f = 0.14 (1:4 EtOAc/Cyclohexane)
R_f = 0.29 (1:3 EtOAc/Cyclohexane)

5-(phenoxymethyl)oxazolidin-2-one (**4d**)
Following the general procedure with 90.1 mg **4** and cyanate (**d**) gave 45 mg crude product (off-white solid). Purification by flash chromatography with EtOAc yielded 26.9 mg **4d**. *Note: The compound is rather poorly soluble in EtOAc and was dissolved and loaded in hot EtOAc.*

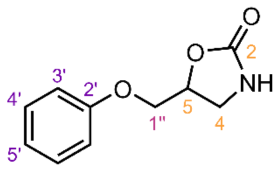

Physical state white, crystalline solid

^{1}H NMR (500 MHz, $CDCl_3$) δ 7.32−7.27 (2H, m, H-4′), 6.99 (1H, tt, *J* = 1.1, 7.4 Hz, H-5′), 6.93−6.89 (2H, m, H-3′), 6.16 (1H, s, N-H-3), 4.96 (1H, ddt, *J* = 4.9, 6.1, 8.8 Hz, H-5) 4.14 (2H, dd, *J* = 1.8, 4.9 Hz, H-1″), 3.77 (1H, dt, *J* = 0.8, 8.8 Hz, H-4), 3.61 (ddd, *J* = 1.0, 6.2, 8.8 Hz, H-4)

^{13}C NMR (125 MHz, $CDCl_3$) δ 159.7 (Cq, C-2), 158.1 (Cq, C-2′), 129.6 (CH, C-4′), 121.6 (CH, C-5′), 114.6 (Cq, C-3′), 74.3 (CH, C-5) 68.0 (CH_2, C-1″), 42.8 (CH_2, C-4)

HRMS m/z calc. 193.074 for $C_{10}H_{11}NO_3$, found 193.074

TLC R_f = 0.02 (1:4 EtOAc/Cyclohexane)

R_f = 0.29 (EtOAc)

1-thiocyanato-3-phenoxypropan-2-ol (**4e.1**), *1-isothiocyanato-3-phenoxypropan-2-ol* (**4e.2**) and potential isomers were obtained and characterized as mixture
Following the general procedure with 90.1 mg **4** and thiocyanate (**e**) gave 64.5 mg crude product (off-white amorphous solid). Purification by flash chromatography with EtOAc yielded 20.2 mg **4e.1+4e.2**.

Physical state white semisolid

^{1}H NMR (500 MHz, $CDCl_3$) δ n.a. due to high signal overlap from the complex mixture

^{13}C NMR (125 MHz, $CDCl_3$) δ n.a. due to high signal overlap from the complex mixture

HRMS m/z calc. 209.050 for $C_{10}H_{11}NO_2S$, found 209.050

TLC R_f = 0.02 (1:4 EtOAc/Cyclohexane)
R_f = 0.28 (EtOAc)

All raw NMR data (^{1}H, ^{13}C, HSQC, and HMBC) are freely available from the externally hosted supplementary information at zenodo.org.[6]

References

(1) Tang, L.; Li, Y.; Wang, X. A High-Throughput Colorimetric Assay for Screening Halohydrin Dehalogenase Saturation Mutagenesis Libraries. *J. Biotechnol.* **2010**, *147* (3), 164–168. https://doi.org/10.1016/j.jbiotec.2010.04.002.
(2) Gul, I.; Fantaye Bogale, T.; Deng, J.; Wang, L.; Feng, J.; Tang, L. A High-Throughput Screening Assay for the Directed Evolution-Guided Discovery of Halohydrin Dehalogenase Mutants for Epoxide Ring-Opening Reaction. *J. Biotechnol.* **2020**, *311*, 19–24. https://doi.org/10.1016/j.jbiotec.2020.02.007.
(3) NMR analysis of 1-chloro-3-phenoxypropan-2-ol (**4f**) and 1-bromo-3-phenoxypropan-2-ol (**4g**) has been reported previously.[4,5]
(4) Martínez, F.; Del Campo, C.; Sinisterra, J.V.; Llama, E.F. Preparation of halohydrin β-blocker precursors using yeast-catalysed reduction. *Tetrahedron: Asymmetry* ***2000***, *11*, 4651–4660. https://doi.org/10.1016/S0957-4166(00)00425-0.
(5) de Andrade, V.S.C.; de Mattos, M.C.S. Trihaloisocyanuric acid/ triphenylphosphine: an efficient system for the regioselective conversion of epoxides into vicinal halohydrins and vicinal dihalides under mild conditions. *Synthesis* **2016**, *48*, 1381–1388. https://doi.org/10.1055/s-0035-1560408.
(6) Kaspar, F. Supplementary Material HHDH. **2024**. https://doi.org/10.5281/zenodo.10785162.
